# Electrostatic Design of the Nanoscale Internal Surfaces of Porous Covalent Organic Frameworks

**DOI:** 10.1021/acs.nanolett.3c00722

**Published:** 2023-04-04

**Authors:** Egbert Zojer

**Affiliations:** Institute of Solid State Physics, NAWI Graz, Graz University of Technology, Petersgasse 16, A-8010 Graz, Austria

**Keywords:** covalent organic framework, metal organic framework, collective electrostatics, level alignment, electronic structure, density-functional theory

## Abstract

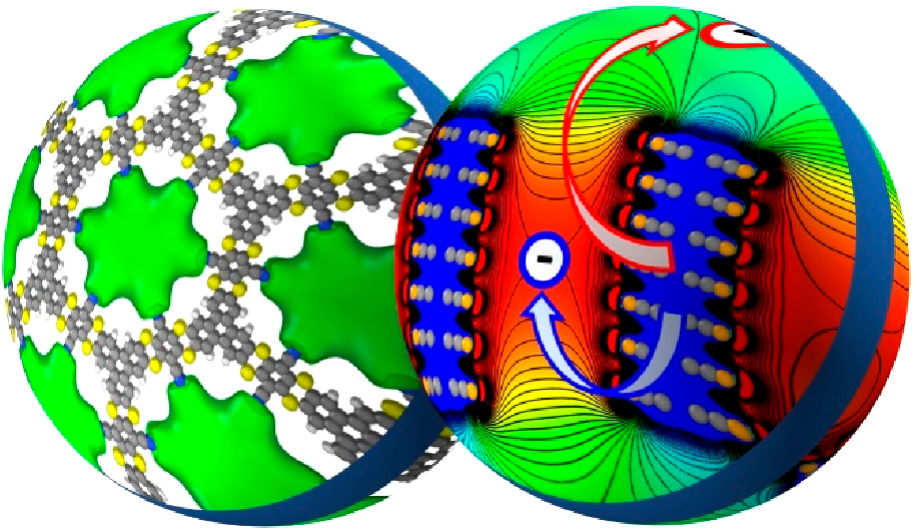

It is well established that the collective action of assemblies of dipoles determines the electronic structure of surfaces and interfaces. This raises the question, to what extent the controlled arrangement of polar units can be used to also tune the electronic properties of the inner surfaces of materials with nanoscale pores. In the present contribution, state-of-the-art density-functional theory calculations are used to show for the prototypical case of covalent organic frameworks (COFs) that this is indeed possible. Decorating pore walls with assemblies of polar entities bonded to the building blocks of the COF layers triggers a massive change of the electrostatic energy within the pores. This, inevitably, also changes the relative alignment between electronic states in the framework and in guest molecules and is expected to have significant impacts on charge separation in COF heterojunctions, on redox reactions in COFs-based electrodes, and on (photo)catalysis.

The electronic structure of flat interfaces, for example, between metals and semiconductors or metals and organic materials, is determined by interfacial charge-transfer processes. The superposition of the fields of the resulting infinitely extended dipole assemblies results in the formation of a step in the electrostatic energy between the region below and the region above the dipoles.^1^ As a consequence of these so-called collective (or cooperative) electrostatic effects,^1−5^ one observes modifications of electrode work-functions,^1−6^ electrostatically induced core-level shifts,^5−7^ massive changes in the ballistic currents through monolayers,^5,8^ and pronounced dependences of thin-film ionization energies on molecular orientation.^9,10^ A controlled application of polar layers at the interface between metal electrodes and organic semiconductor materials can also change contact resistances by several orders of magnitude and even switch the polarity of the transported charges.^6,11^ In the context of metal organic and covalent organic frameworks (MOFs and COFs), periodic assemblies of polar groups have been suggested in order to tune the electronic states within the framework structures with the goal to either localize charge carriers^12^ or to induce extended potential gradients.^13^ First steps toward implementing these ideas in actual systems have already been made.^14,15^ This raises the question, whether the controlled assembly of polar entities could also be straightforwardly employed for changing the electronic properties of internal surfaces in porous materials like COFs, MOFs, or zeolites.

In the following, this question will be addressed for the prototypical case of layered two-dimensional (2D) COFs stacked in a (close to) eclipsed fashion such that open, one-dimensional (1D) channels are formed. In general, COFs have attracted a lot of interest in recent years due to their potential in a variety of fields^16,17^ including photocatalyis,^18^ electro-catalysis,^19^ energy storage,^20−25^ optoelectronics (including sensing),^26−28^ and photovoltaics.^29−32^ In the present context they are interesting primarily, because their channels can be decorated by polar groups pointing toward the pore centers in a relatively straightforward manner, for example, through substituting the COF building blocks with polar groups (Figure 1). Such polar substituents are rather common in COFs,^17^ with polar entities comprising, e.g., nitrile groups (e.g., in COF-316,^33^ JUC-505,^34^ or DUT-177^25^ – for the latter see Figure 1a), ketones (e.g., in DAAQ-TFP^20^ and in JUC-506^34^), or halogens (e.g., in COF-F^35^). Notably, the said polar groups are frequently altered in postsynthetic modification reactions.^25,33^ This typically changes the magnitude of the dipoles and sometimes even their direction (e.g., when converting nitriles to amides or amidoximes,^33^ to amines,^34^ or to polysulfides^25^). A similar situation prevails, when switching the polarity of the substituents in the course of redox reaction, e.g., when converting ketones to alcohols.^20^

**Figure 1:**
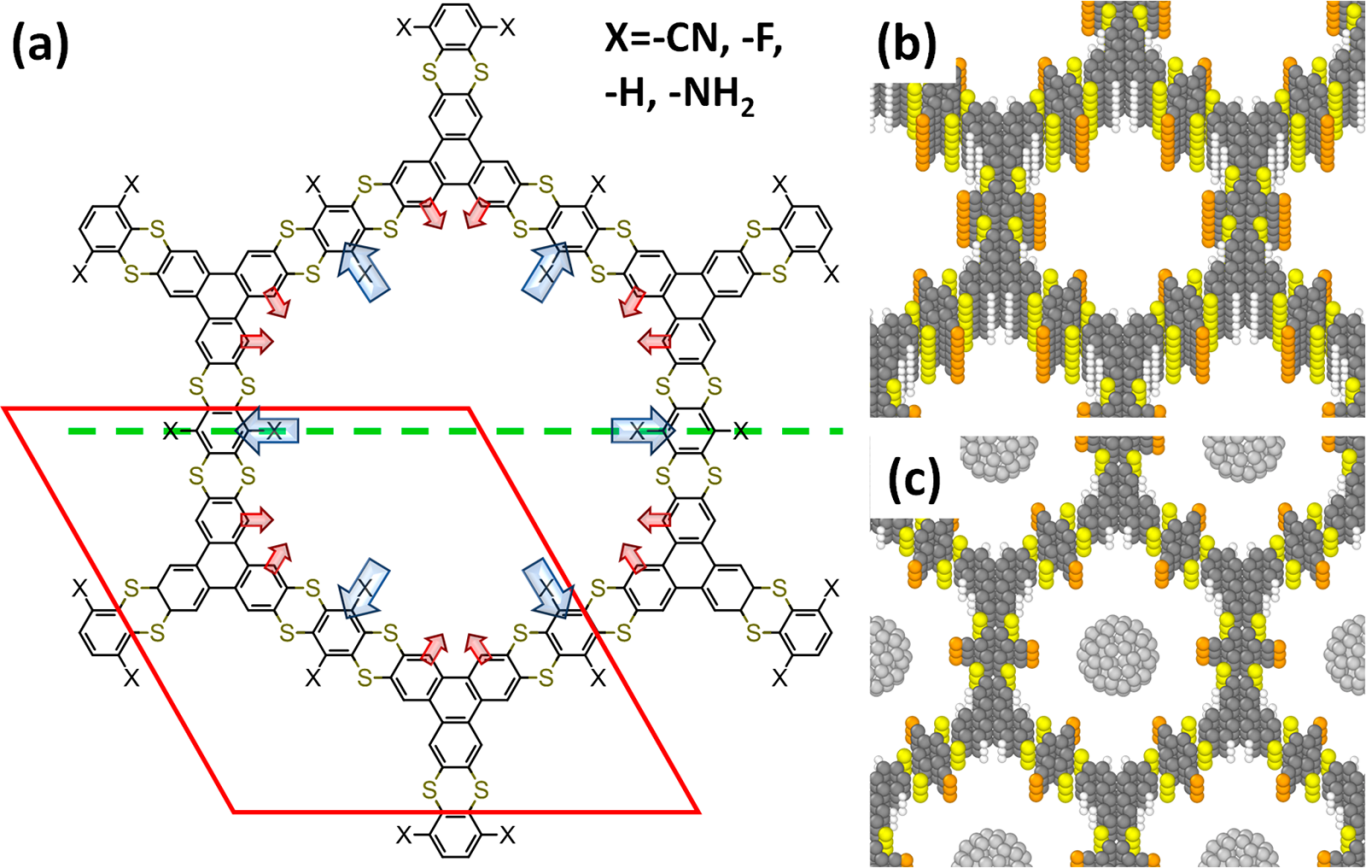
(a) Chemical structures of the studied COFs derived from DUT-177 (the structure with X = -CN); the red diamond represents the unit-cell of the structure; the blue arrows denote the dipoles of the substituents (here for X = -CN; pCOF-CN). The red arrows highlight the smaller, inward-pointing dipoles due to the C–H bonds. The dashed green line indicates the plane for which cross sections of the electrostatic energy are plotted in Figure 3. (b) Structure of a 7-layer slab of pCOF-CN; the lateral extent of the unit-cell in these calculations is the same as in panel a, while in the vertical direction the unit cell contains a fixed number of COF sheets. (c) Structure of the MOF containing C_60_ in the center of the pores to explicitly assess the alignment of electronic levels between the COF and guest molecules (example of pCOF-CN); the pCOF-C_60_ structure is a continuous bulk structure (see section S7 in the Supporting Information) and contains one C_60_ molecule per pore per three layers of COF. (Color code – dark gray: C in the COF, light gray: C in C_60_, yellow: S, orange: N, white: H; plots produced using Marvinsketch (https://www.chemaxon.com) and OVITO.^42^)

In the present study it will be clarified, (i) whether dipole-induced effects in layered COFs cause significant shifts between the electrostatic energy inside the pores, the electronic states of the COF material, and the vacuum level defined outside the sample; (ii) whether the nature of such shifts is collective, i.e., whether it is a direct consequence of the periodic arrangement of the dipoles in the channels; (iii) whether the shifts can be modified or even inverted by modifying the polarity of the substituents that decorate the pores; and (iv) whether collective electrostatic shifts could be used to tune the energy-level alignment within COFs containing guest molecules. A handle to tune that level alignment would be of distinct relevance, for example, for charge separation in guest–host type COF heterojunctions,^28−30,36,37^ for redox-active COFs used, e.g., as battery electrodes,^25,38^ and for (photo)catalysis applications.^39,40^

Addressing the above questions requires an understanding of the materials at an atomistic level, which for complex systems can be best achieved by means of state-of-the-art dispersion-corrected density-functional theory calculations. Such simulations have frequently been applied to accurately describe collective electrostatic effects at flat surfaces and to obtain qualitative and also quantitative agreement between experiments and simulations.^41^

The starting point for the following considerations is DUT-177, a COF first introduced in ref. (25), which is conceptually similar to COF-316.^33^ It consists of essentially 2D layers formed by thianthrene-based elements, as shown in Figure 1a (DUT-177 is the structure with -X = -CN). Due to the buckled conformation of the COF building blocks, these layers are not entirely flat. Moreover, as shown in ref. (25), consecutive layers of DUT-177 are stacked in a slightly serrated manner. To ease the construction of suitable model systems, in the present study, an eclipsed stacking is enforced (see Figure 1b). This, however, has no noticeable impact on the effects discussed here, as shown in section S3 of the Supporting Information. To test the role of the polarity of the substituents facing the pore walls, the -CN substituents in the X-positions of DUT-177 have been replaced by -F, -H, and -NH_2_. For the sake of consistency, these systems in the following will be referred to pCOF-CN (=DUT-177), pCOF-F, pCOF-H, and pCOF-NH_2_ (where pCOF stands for polar covalent organic framework).

Given the letter character of the paper, a detailed description of all methodological aspects is provided in section S2 in the Supporting Information. In short, the bulk structures of all systems were fully optimized using the FHI-aims code^43−45^ employing the PBE functional^46,47^ and a nonlocal variant of the many-body dispersion correction.^48^ To locally probe the electrostatic potential, electrostatic core-level shifts^7^ for suitably placed, inert Ne atoms were used, yielding results fully consistent with the calculated Hartree energies.

Based on the optimized geometries, a variety of model systems were constructed (for details see sections S1 and S2 of the Supporting Information): (i) Systems with an artificially increased stacking distance (Figure S1) were used to assess the collective nature of the observed effects. They were realized by increasing the length of the **a**_**3**_ unit-cell vector. Systems with a stacking distance increased by 50 Å served as converged models for isolated monolayers. (ii) To allow for an unambiguous definition of a reference energy (see below), also finite thickness slabs containing up 11 COF layers were studied, separating the electrostatically decoupled^49,50^ periodic replicas of the slabs by a 70 Å vacuum region. (iii) Finally, to determine the impact of collective electrostats on the energy alignment between electronic states in the COFs and in guest molecules, C_60_ molecules were placed inside the pores, as shown in Figure 1c (see also section S7 of the Supporting Information). C_60_ is chosen here, as together with its derivatives it is the prototypical system for exploiting excited-state charge transfer processes in organic and hybrid systems and because it has repeatedly been considered as guest molecule in COF channels.^29,30,36,37^ Notably, the insights derived from the COF/C_60_ hybrid material bear direct relevance also for any other situation in which charge-transfer processes between guests and the COF backbone play a role.

The electrostatic energy in the center of the pore relative to the situation of the isolated monolayer, Δ*E*_*pore*_^*elstat*^, is shown for all studied pCOFs in Figure 2a as a function of the stacking distance. The plot reveals a massive shift of the electrostatic energy when the interlayer distance is decreased. It amounts to ∼0.8 eV for pCOF-CN and to ∼−0.5 eV for pCOF-NH_2_, i.e., there is a significant impact of nearby layers on the electrostatic energy in the pores and the sign of the shift clearly depends on the polarity of the substituents. Both observations are a clear indication of a strong collectivity of the observed shift and of its origin being electrostatic. This assessment is confirmed by the data for the two substituents with reduced polarity, pCOF-F and pCOF-H. The somewhat surprising observation that for pCOF-F the absolute value of Δ*E*_*pore*_^*elstat*^ is vanishingly small, is a consequence of the additional dipoles due to the C–H bonds facing the channel and occurring in all COFs (see red arrows in Figure 1a). They induce a shift to smaller values of Δ*E*_*pore*_^*elstat*^, which due to the large number of C–H dipoles facing the channel is barely compensated by the oppositely oriented C–F dipoles. While the analysis of Δ*E*_*pore*_^*elstat*^ testifies to the possibility to manipulate the potential in the pore via electrostatic effects, it does not allow to fully quantify the effect. This has two reasons: First, calculating Δ*E*_*pore*_^*elstat*^ does not allow assessing the impact of the dipoles within an isolated monolayer, as the latter serves as the reference. Second, it is impossible to define a unique and unambiguous reference point for the energy scale that stays constant for all systems at all layer distances. This becomes obvious in Figure 2a when comparing the electrostatic energies in the pores relative to the energies of the valence-band maxima (VBM; open symbols) and relative to the average of the highest and lowest C-1s binding energies of atoms within the COF backbone (C_1s-average_; filled symbols), as two of the obvious energy references for a bulk system. Unfortunately, the positions of these references change with layer distance due to local fields and band broadening effects (see below).

**Figure 2:**
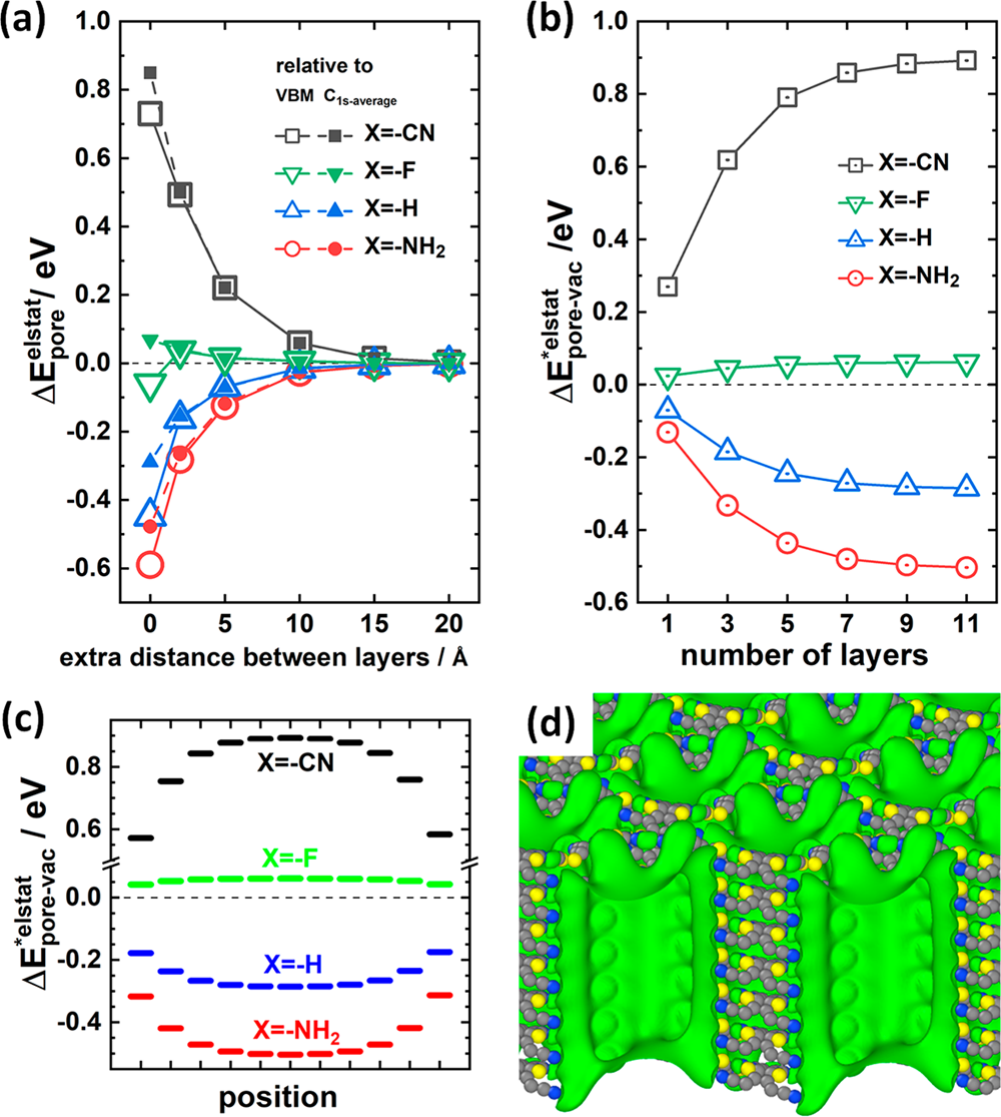
Electrostatic energy inside the pores of pCOFs: (a) Electrostatic energies in the center of the pore relative to the value for the isolated monolayer approximated by a system with a spacing between COF layers increased by 50 Å, Δ*E*_*pore*_^*elstat*^, plotted as a function of the distance between the COF layers for a fully periodic structure. 0 Å refers to the equilibrium distance (corresponding to a unit-cell height between 3.92 Å in the case of pCOF-CN and 3.86 Å for pCOF-NH_2_). Δ*E*_*pore*_^*elstat*^ is calculated as the difference between the electrostatic energy at the center of the pore for a given distance, *E*_*pore*_^*elstat*^, and the electrostatic energy for an extra spacing of 50 Å, *E*_*pore*,50_^*elstat*^ (Δ*E*_*pore*_^*elstat*^ = *E*_*pore*_^*elstat*^ – *E*_*pore*,50_^*elstat*^). Notably, the determination of the energies require a definition of the zero of the energy scale in each of the considered systems (as an absolute energy scale does not exist). The open symbols refer to the situation when choosing the valence-band maximum in the respective systems as the energy reference, while the filled symbols describe energies relative to the mean of the highest and lowest C-1s core-level binding energies. (b) Electrostatic energies for finite thickness slabs determined as the difference between the electrostatic energy in the middle of the pore of the central layer and the electrostatic energy in a vacuum 30 Å above the stack, *E*_*pore*-vac_^*elstat^, as a function of the number of layers in the stack. Panel (c) shows the electrostatic energy in the center of the pore for each COF layer in the 11-layer stacks, *E*_pore_^*elstat^, relative to the electrostatic energy in a vacuum, *E*_vac_^*elstat^, with Δ*E*_*pore*-vac_^*elstat^ = *E*_pore_^*elstat^ – *E*_vac_^*elstat^. Panel (d) contains an isovalue plot of the electrostatic energy for a pCOF-CN 7-layer stack. The isovalue has been set to 0.7 eV relative to the vacuum level. All data used to generate the plots in panels (a)–(c) are contained in Table S3 in the Supporting Information. OVITO^42^ was used for the generating the isovalue plot.

To mend this problem, also stacks of pCOFs comprising finite numbers of layers were investigated. Such calculations provide access to the vacuum level above the slabs as a unique and system-independent reference energy. First, calculations confirm the assessment that the position of the CBM does not qualify as a reliable energy reference, as when comparing the 1- and 11-layer slabs the CBM energy relative to the vacuum level is lowered by 0.09 eV for pCOF-CN and raised by 0.21 for pCOF-NH_2_. Moreover, using the vacuum level as an energy reference provides access to the arguably most relevant quantity describing the dipole-induced effects, namely the difference between the electrostatic energy in the center of the pore (at the position of the central pCOF layer) and the electrostatic energy outside the COF, *E*_*pore*-vac_^*elstat^. Here, the * denotes the fact that one is now dealing with a system of finite thickness. The values of *E*_*pore*-vac_^*elstat^ are shown in Figure 2b as a function of the number of layers in the slab, N. One observes a pronounced dependence of *E*_*pore*-vac_^*elstat^ on N, with values for pCOF-CN starting at +0.27 eV for the monolayer and saturating at +0.89 eV for the 11-layer stack. For pCOF-NH_2_ the values vary between −0.13 eV and −0.50 eV. This supports the notion that the shift is collective in nature, as it increases with the number of neighboring layers. The collectivity is further confirmed by the data in Figure 2c, where the evolution of *E*_*pore*-vac_^*elstat^ is shown as a function of the position along the pore axis revealing pronounced edge effects.

Second, the data in Figure 2b show that the magnitude of the dipole-induced variation of the electrostatic energy within the pores is sizable. The energy difference between the two most extreme COFs studied here amounts to as much as 1.39 eV. At this point it needs to be stressed that the data in Figure 2b represent the situation in the pore center at a considerable distance from the atoms constituting the COF backbone. Thus, the observed differences are not a consequence of chemical “through-bond” substitution effects. As discussed below, these mostly affect the positions of the electronic states within the COFs relative to the vacuum level. Rather, Figure 2b illustrates the situation within the pore that is caused by a superposition of the electric fields originating from the polar groups decorating the pore walls.

The formation of a “potential-pocket” within the pore is illustrated in Figure 2d, which shows an isovalue plot for the electrostatic energy at +0.7 eV above the vacuum level for the 7-layer stack of pCOF-CN. Notably, the “potential-pocket” comprises essentially the entire pore region. Variants of such plots for all systems are discussed in section S6 in the Supporting Information. As an alternative visualization approach, cross sections of the electrostatic energy landscapes within the pores of the four studied systems are shown in Figure 3. The plots confirm the massive increase in electrostatic energy within the pore of pCOF-CN, indicating even higher energy values, when moving from the pore center toward the substituents (see isolines). Only in the immediate vicinity of the atoms forming the COF, the electrostatic energy becomes negative (blue) due to the electrostatic potential of the nuclei. The drop in electrostatic energy upon approaching the surface of the stack (inferred already from Figure 2c) is also clearly resolved. For pCOF-F in Figure 3b, one sees that the increase of the electrostatic energy is confined to the immediate vicinity of the F atoms, while for pCOF-H (Figure 3c) and pCOF-NH_2_ (Figure 3d), the electrostatic energy is significantly lower inside the pore than in the vacuum region above the stack. This is fully consistent with the data in Figure 2b.

**Figure 3:**
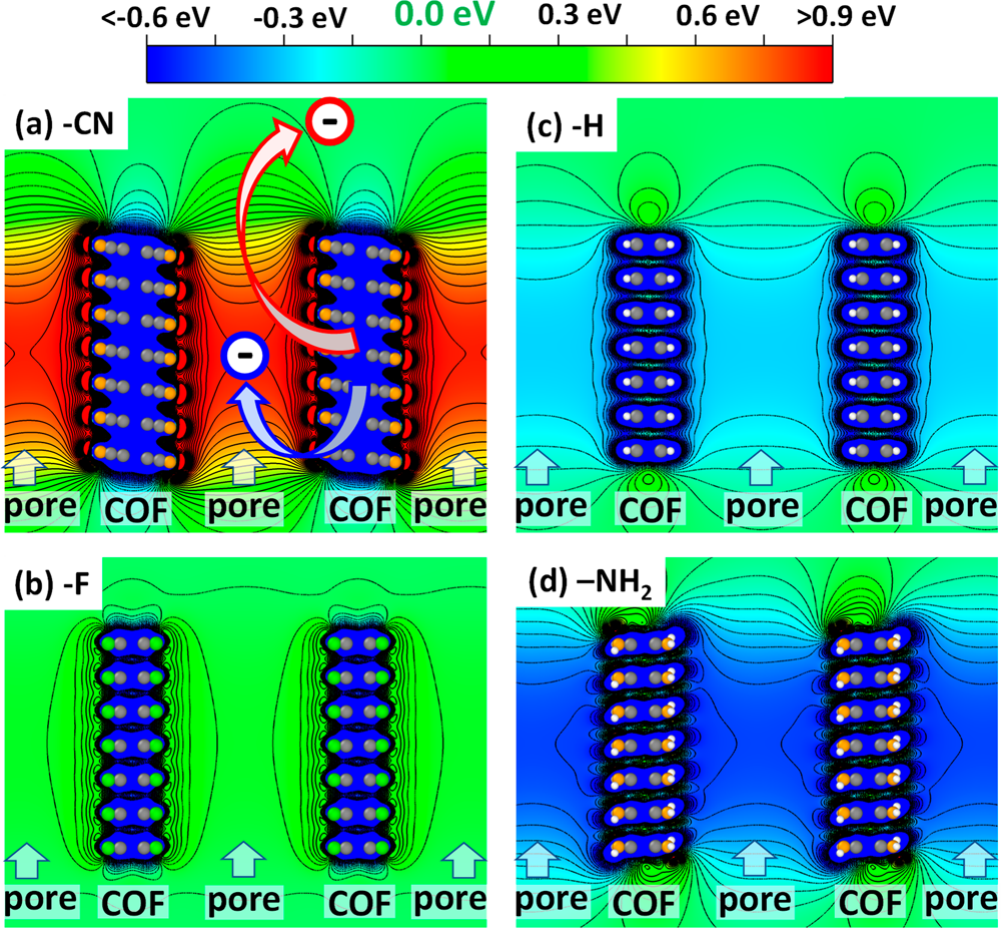
Center of pore electrostatic energy (Hartree energy) relative to the vacuum energy for 7-layer stacks of pCOF-CN (a), pCOF-F (b), pCOF-H (c), and pCOF-NH2 (d). Isolines are drawn between −1.0 and 1.5 V every 0.05 eV; The planes for which the potentials are plotted have been chosen such that they cut through the centers of the pores. Their positions are indicated by the dashed green line in Figure 1a. The structures of the COFs are overlaid for the sake of clarity and the vertical regions associated with pores and COF backbones are indicated. In panel (a), also the fundamental difference between injecting an electron from the COF into the interior of the pore and into vacuum is illustrated (plots produced using VESTA^51^).

The above results raises the question: what would be the practical impact of the dipole-triggered change in electrostatic energy inside the pore? An obvious consequence should be a largely rigid shift of all electronic states of any species contained inside the pores relative to the electronic states in the pCOF. To test that hypothesis, we studied (infinitely extended) pCOFs containing C_60_ molecules in the centers of the pore (for details, see above and section S7 in the Supporting Information). The calculated densities of states projected onto the COF and onto the C_60_ guests are shown for the four studied systems in Figure 4a. Indeed, one observes a significant shift of the states in C60 to lower energies relative to the bands in the COF for the series pCOF-CN → pCOF-F → pCOF-H → pCOF-NH_2_. This shift is exemplarily highlighted for the lowest unoccupied band. In fact, for pCOF-NH_2_ that band is shifted so much that it is pinned at the VB of the COF, which triggers a ground-state charge transfer and limits the overall magnitude of the shift in that system. It should be mentioned that this specific observation might well be a consequence of the notoriously too small band gap caused by the many-electron self-interaction error inherent to (semi)local functionals. In passing it is noted that conventional hybrid functionals could potentially reduce (albeit certainly not fix)^52^ the band gap problem, but at nonaffordable computational costs for the huge systems studied here. Moreover, the potentially spurious charge transfer in the pCOF-NH_2_, does not compromise the overall effect of a significant change of the level alignment.

**Figure 4:**
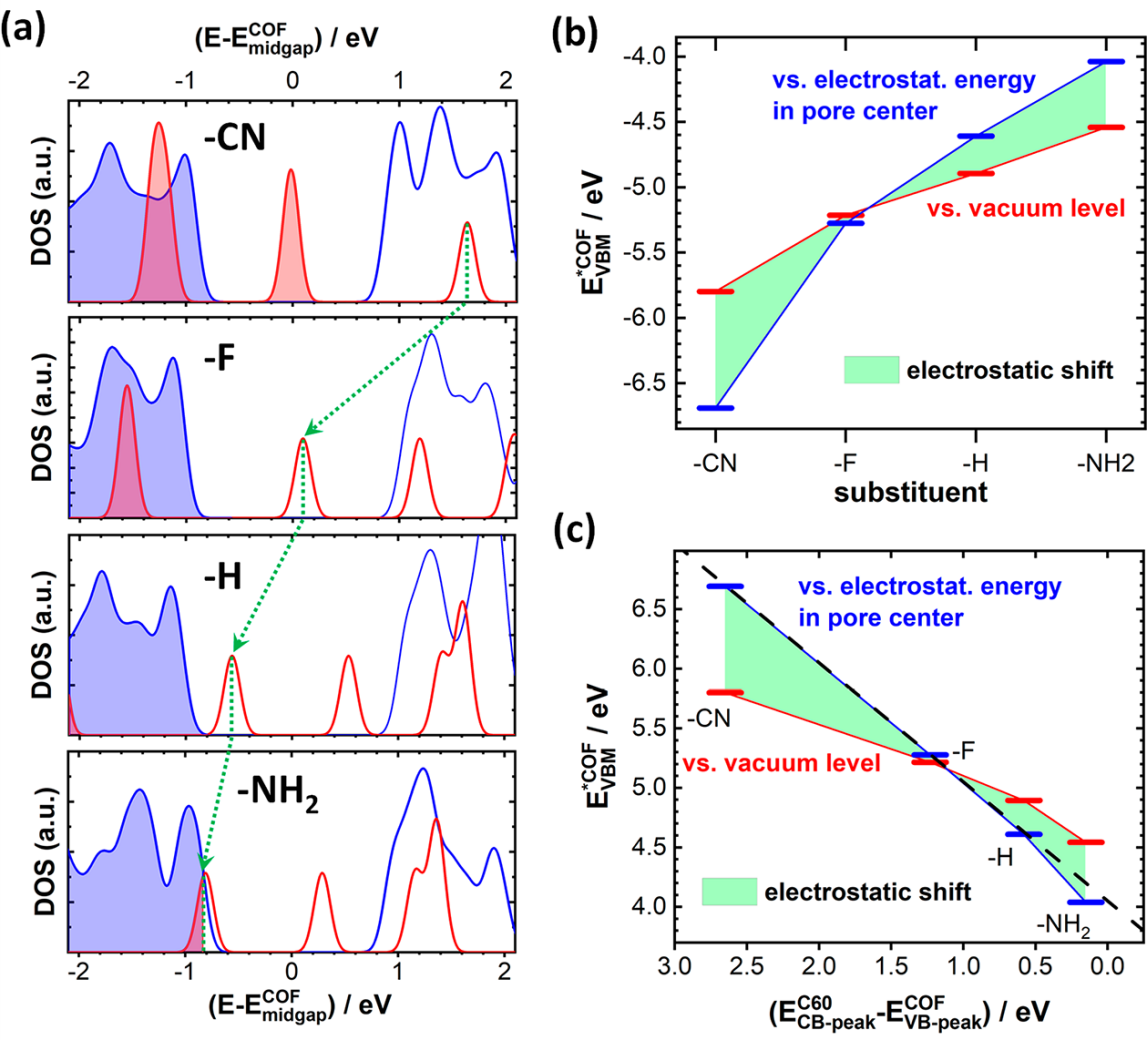
(a) Densities of states of pCOF-CN, pCOF-F, pCOF-H, and pCOF-NH_2_ (blue lines) and of C_60_ molecules (red lines) in structures equivalent to those shown in Figure 1c. The energy scale is aligned to the center of the band gap of the respective COFs, *E*_*midgap*_^*COF*^. The (non) shaded states in the DOSs are occupied (unoccupied). The dotted green lines/arrows connect the positions of the DOS peaks associated with the conduction bands of the C_60_ chains and serve as a guide to the eye. (b) Red bars: substitution dependence of the energy of the valence-band maximum of the COF, *E*_VBM_^*COF^, relative to the electrostatic energy in a vacuum. This energy correlates with the ionization process denoted by the red arrow in Figure 3(a). Within the single-electron picture, it corresponds to the ionization-energy of the COF. Blue bars: substitution dependence of the energy of the valence-band maximum relative to the electrostatic energy in the center of the pore of the middle COF layer of a COF stack (charge-transfer process represented by the blue arrow in Figure 3(a)). The displayed data were obtained for the 11-layer stack, for which the electrostatic energy in the central COF layer has essentially converged to the bulk value (see Figure 2b). The area shaded in green corresponds to the “additional” shift due to the collective electrostatic modification of the potential inside the pore. An equivalent plot for the conduction-band minimum is contained in Figure S9 in the Supporting Information. (c) Plot similar to (b), but now the energy differences, *E*_VBM_^*COF^, are plotted as a function of the offset between peaks in the DOSs associated with the valence band of the COFs, *E*_*VB-peak*_^*COF*^, and the conduction band of the C60 chains, *E*_*CB-peak*_^*C*60^. The dashed black line has a slope of 1 and serves as guide to the eye. It illustrates what reference energy for *E*_VBM_^*COF^ determines the level alignment between the COF and the C60 states.

At this point, one might argue that the different level alignment between the states in the COFs and in C_60_ could be merely the consequence of classical chemical substitution effects due to attaching electron donating or accepting groups to the COF backbones. Such chemical shifts change the global ionization energy and electron affinity, and, thus, (in a single particle picture) shift the positions of the valence band maximum and the conduction band minimum (CBM) of the COF relative to the vacuum level. For the VBM the corresponding ionization process is illustrated by the red arrow in Figure 3a. The system-dependent evolution of the energy of the VBM of the COF, *E*_VBM_^*COF^, relative to the vacuum level is illustrated for the 11 layer stacks by the red bars in Figure 4b. As expected, one observes an appreciable chemically induced shift, but when instead plotting the position of the VBM relative to the electrostatic energy inside the pore (blue bars in Figure 4b), the obtained shift is much larger. The reason for that is that when considering the ionization/charge transfer process indicated by the blue arrow in Figure 3a, the collective electrostatic shifts discussed above (highlighted by the green shading in Figure 4b) are superimposed on the chemical ones and strongly amplify their effect. The crucial question is, whether the actual level alignment of the C_60_ and COF states from Figure 4a, is determined by the position of the VBM of the COF relative to the vacuum energy above the sample (red bars) or relative to the electrostatic energy in the pores (blue bars).

To answer that, Figure 4c plots the trend in *E*_VBM_^*COF^ (both, relative to the vacuum level and relative to the electrostatic energy in the pore) as a function of the level alignment in the COF/C_60_ guest–host system. The latter is quantified by the energetic offset between the DOS peaks associated with the conduction band of C60 and the valence band of the respective COF. When considering only chemical shifts, the substitution-dependent variation of *E*_VBM_^*COF^ is far smaller than the shift in the level alignment, as can be inferred from the deviations of the red bars from the dashed black line (which has a slope of 1). Only, when additionally considering dipole-induced shifts in the electrostatic energy of the pore (blue bars), a one-to-one correspondence between the evolution of *E*_VBM_^*COF^ and the change in level alignment is obtained (with a minor deviation for pCOF-NH_2_ due to the above-described pinning). This clearly shows that collective electrostatic shifts due to a decoration of pore-walls with polar groups can massively impact the alignment between the electronic states in the COFs and in adsorbates, with consequences for a wide variety of processes. In the present case, they double the change in level alignment due to the substituents.

The above considerations show that decorating the walls of nanoscale pores by ordered assemblies of polar units can massively change the electrostatic energy within the pores and the electronic structure of guest–host systems. This is explicitly shown for stacks of 2D COF layers made from building blocks containing polar substituents pointing toward the pore centers. More specifically, the present study focuses on derivatives of the recently introduced DUT-177 system,^25^ in which the original -CN substituents were systematically replaced by -F, -H, and -NH_2_ groups. This allows varying the electrostatic energy within the pores relative to the vacuum energy outside the COF by as much as 1.4 eV. That effect is collective in nature, i.e., it is largely a consequence of the superposition of the fields of all dipoles contained in the walls of the channels. This is shown by systematically varying the interlayer distance and the number of stacked COF layers, while following the evolution of the electrostatic energy within the pores.

A key consequence of the dipole-induced variation of the electrostatic energy is that incorporating polar groups into the pore walls allows tuning the energetic alignment of electronic states in the COF host relative to states on guest molecules contained in the pores. This is explicitly shown for C_60_ guest molecules, which are often infiltrated into pores for realizing charge separation in guest–host type COF heterojunctions.^28−30,36,37^ Here the alignment of the valence-band maximum of the COF and the conduction-band minimum of the guests changes by at least 2.4 eV between pCOF-CN and pCOF-NH_2_. It can be shown that less than half of that shift originates from the “chemical” effect due to replacing electron accepting substituents by electron donating ones. The somewhat larger contribution to the shift can be traced back to the collective shift of the electrostatic energy in the channels caused by the periodic arrangement of the dipoles.

For the incorporation of C_60_ molecules (as classical electron acceptors in organic solar cells), this is expected to have a considerable impact on excited state charge transfer processes. Beyond that, the electrostatic modification of the energy level alignment is expected to also significantly influence redox processes in battery applications and the (photo)catalytic efficiency of COFs, as also in these applications the alignment of energy levels plays a decisive role.^38−40^ In passing, we note that the results presented here also imply that postsynthetic modification reactions that change the dipole moment or polarity of the substituents^20,25,33,34^ will diminish or enhance the beneficial collective electrostatic effects mentioned above and can, therefore, have consequences for the functionality of a COF far beyond the original intention for performing the modification.

Finally, due to their electrostatic nature, none of the effects discussed above are restricted to 2D COFs but apply to porous materials in general. All the above arguments suggest that the design of the energy landscape of porous materials and guest–host systems exploiting collective electrostatic effects is absolutely crucial for optimizing functional porous systems.

## Data Availability

The data underlying this study are available from the NOMAD repository (DOI: 10.17172/NOMAD/2023.03.23-19).

## Supporting Information Available

Details on the investigated structure and on the employed modeling strategy; comparison of eclipsed and serrated COF structures, tabulated Ne-1s core level energies for determining shifts in electrostatic energies i, a variety of isovalue plots of the electrostatic energy for periodic COFs and COF stacks, and additional information on C60 containing COFs (PDF)
