# Supplementary material for: Electrostatic Design of the Nanoscale Internal Surfaces of Porous Covalent Organic Frameworks
Source: Nano Lett. 2023 Apr 4;23(8):3558–64. doi: 10.1021/acs.nanolett.3c00722 (PMC10141416; doi:10.1021/acs.nanolett.3c00722)
Supplement: Supplementary file 1 — nl3c00722_si_001.pdf [file nl3c00722_si_001.pdf]

*Supporting Information for*

# Electrostatic design of the nano-scale internal surfaces of porous covalent organic frameworks

Egbert Zojer

*Institute of Solid State Physics, NAWI Graz, Graz University of Technology, Petersgasse 16, A-8010 Graz, AUSTRIA*

**Data Availability:** The data underlying this study are available from the NOMAD repository using the following DOI: 10.17172/NOMAD/2023.03.23-19.

**Table of contents:**

S1. Structural details of the investigated structures 2

S2. Additional methodological details 4

S2.1 computational approach 4

S2.2 Determining the electrostatic energy 6

S2.3 Details on the employed basis set 6

S2.4. Impact of basis-set and k-point sampling 7

S3. Eclipsed vs. serrated structure 8

S4. Additional details on the determination of the vacuum level 9

S5. Core level binding energies and their shifts for the Ne atoms used as probes for the electrostatic potential 10

S6. Isovalue plots of the electrostatic energies for selected periodic COFs and COF stacks 14

S7. Additional information on the simulation of COFs containing C60 molecules in the pores 18

# S1. Structural details of the investigated structures

For pCOF-CN, two types of structures were considered: Structures in which an eclipsed stacking was assumed (i.e., structures in which the unit-cell vector $\mathbf{a_3}$ was fixed to be orthogonal to $\mathbf{a_1}$ and $\mathbf{a_2}$ and in which the angle between $\mathbf{a_1}$ and $\mathbf{a_2}$ was fixed to 60°) and structures with a somewhat serrated conformation as described also in [1], in which all unit-cell parameters were optimized (with a slightly distorted structure as starting geometry to avoid getting stuck in a saddle point during the optimization). This yielded the following unit-cell vectors. More digits can be found in the datasets uploaded to the NOMAD database.

*Table S1: Unit-cell parameters of the studied systems*

pCOF-CN eclipsed structure:

$\mathbf{a_1}$=(22.666, 13.086, 0.000) Å, $\mathbf{a_2}$=(-22.257, 12.850, 0.000) Å, $\mathbf{a_3}$=(0.000, 0.000, 3.918 Å)

$a_1$= 26.171 Å, $a_2$= 25.700 Å, $a_3$= 3.918 Å, angle($\mathbf{a_1}$,$\mathbf{a_2}$)=120.0°, angle($\mathbf{a_2}$,$\mathbf{a_3}$)=90.0°, angle($\mathbf{a_1}$,$\mathbf{a_3}$)=90.0°

pCOF-CN serrated structure (fully optimized):

$\mathbf{a_1}$=(22.508, 12.956, 0.048) Å, $\mathbf{a_2}$=(-22.533, 12.675, 0.013) Å, $\mathbf{a_3}$=(0.379, 0.392, 3.894 Å)

$a_1$= 25.971 Å, $a_2$= 25.853 Å, $a_3$= 3.918 Å, angle($\mathbf{a_1}$,$\mathbf{a_2}$)=120.7°, angle($\mathbf{a_2}$,$\mathbf{a_3}$)=92.0°, angle($\mathbf{a_1}$,$\mathbf{a_3}$)=82.2°

pCOF-F eclipsed structure:

$\mathbf{a_1}$=(22.412, 12.939, 0.000) Å, $\mathbf{a_2}$=(-22.358, 12.908, 0.000) Å, $\mathbf{a_3}$=(0.000, 0.000, 3.886 Å)

$a_1$= 25.879 Å, $a_2$= 25.816 Å, $a_3$= 3.918 Å, angle($\mathbf{a_1}$,$\mathbf{a_2}$)=120.0°, angle($\mathbf{a_2}$,$\mathbf{a_3}$)=90.0°, angle($\mathbf{a_1}$,$\mathbf{a_3}$)=90.0°

pCOF-H eclipsed structure:

$\mathbf{a_1}$=(22.476, 12.977, 0.000) Å, $\mathbf{a_2}$=(-22.327, 12.890, 0.000) Å, $\mathbf{a_3}$=(0.000, 0.000, 3.921 Å)

$a_1$= 25.953 Å, $a_2$= 25.781 Å, $a_3$= 3.918 Å, angle($\mathbf{a_1}$,$\mathbf{a_2}$)=120.0°, angle($\mathbf{a_2}$,$\mathbf{a_3}$)=90.0°, angle($\mathbf{a_1}$,$\mathbf{a_3}$)=90.0°

pCOF-$NH_2$ eclipsed structure:

$\mathbf{a_1}$=(22.249, 12.845, 0.000) Å, $\mathbf{a_2}$=(-22.384, 12.924, 0.000) Å, $\mathbf{a_3}$=(0.000, 0.000, 3.864 Å)

$a_1$= 25.691 Å, $a_2$= 25.847 Å, $a_3$= 3.864 Å, angle($\mathbf{a_1}$,$\mathbf{a_2}$)=120.0°, angle($\mathbf{a_2}$,$\mathbf{a_3}$)=90.0°, angle($\mathbf{a_1}$,$\mathbf{a_3}$)=90.0°

The slightly different lengths of the $\mathbf{a_1}$ and $\mathbf{a_2}$ vectors are attributed to the non-planar conformation of the COF layers and to the specific choice of the initial tilts of the various building blocks of the COF. Notably,

different unit-cell vector lengths were reported in [1]. In this context it should be noted that structural details like that do not have any impact on the effects reported in this paper (see also below).

The following two figures illustrate the systems studied when maintaining a fully periodic bulk structure, but increasing the inter-layer distance (Figure S1) and when studying finite-thickness stacks employing the repeated-slab approach (Figure S2).

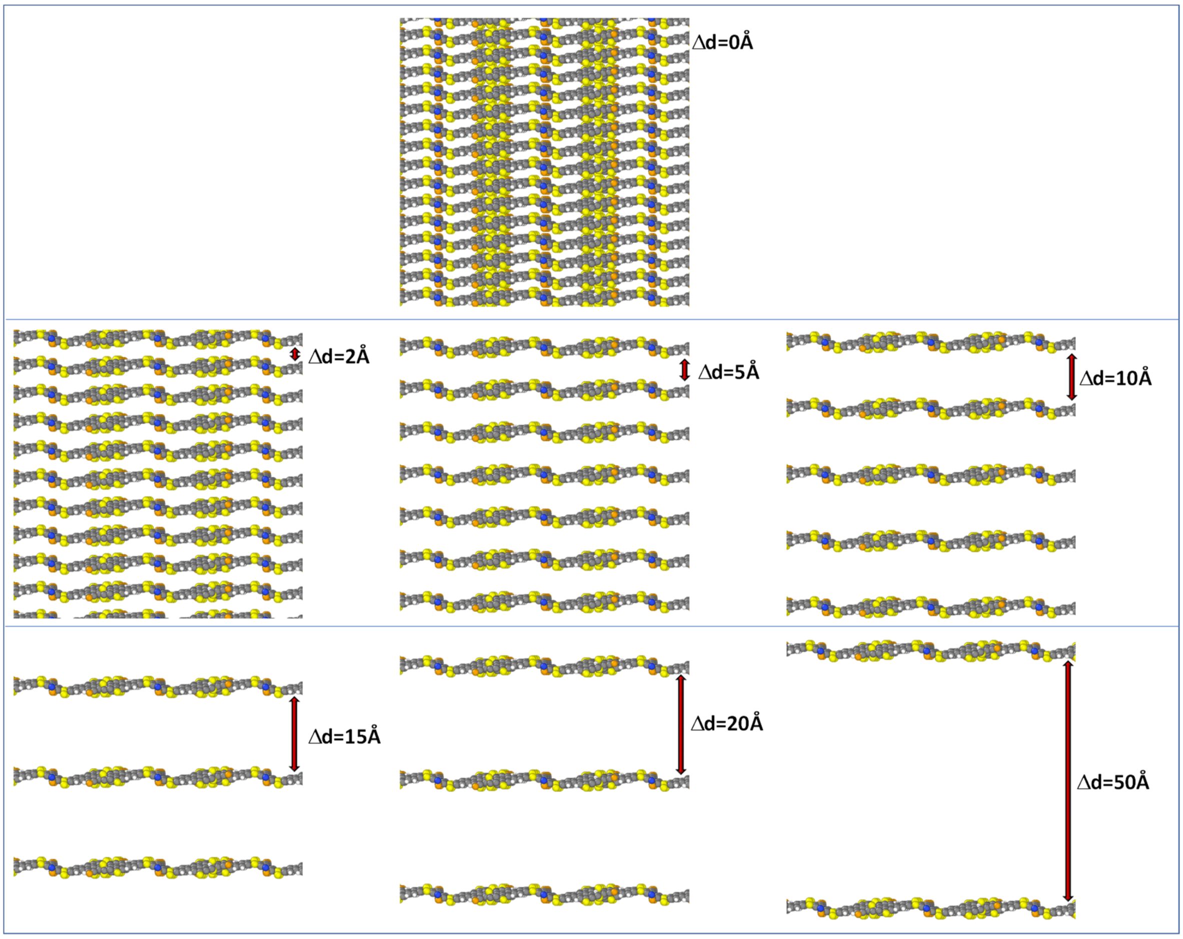


*Figure S1: Side views of the periodic COFs with varying inter-layer distance. The top panel shows the equilibrium structure and in the following panels the structures obtained when increasing the inter-layer distance by Δd are shown. As for the displayed structure an eclipsed packing has been assumed, Δd also corresponds to the value by which the lattice constant a3 has been increased. The atomic positions have been optimized only for the densely packed structures. (color code - dark grey:C in the COF, light grey: C in C60, yellow: S, orange: N, white: H)*

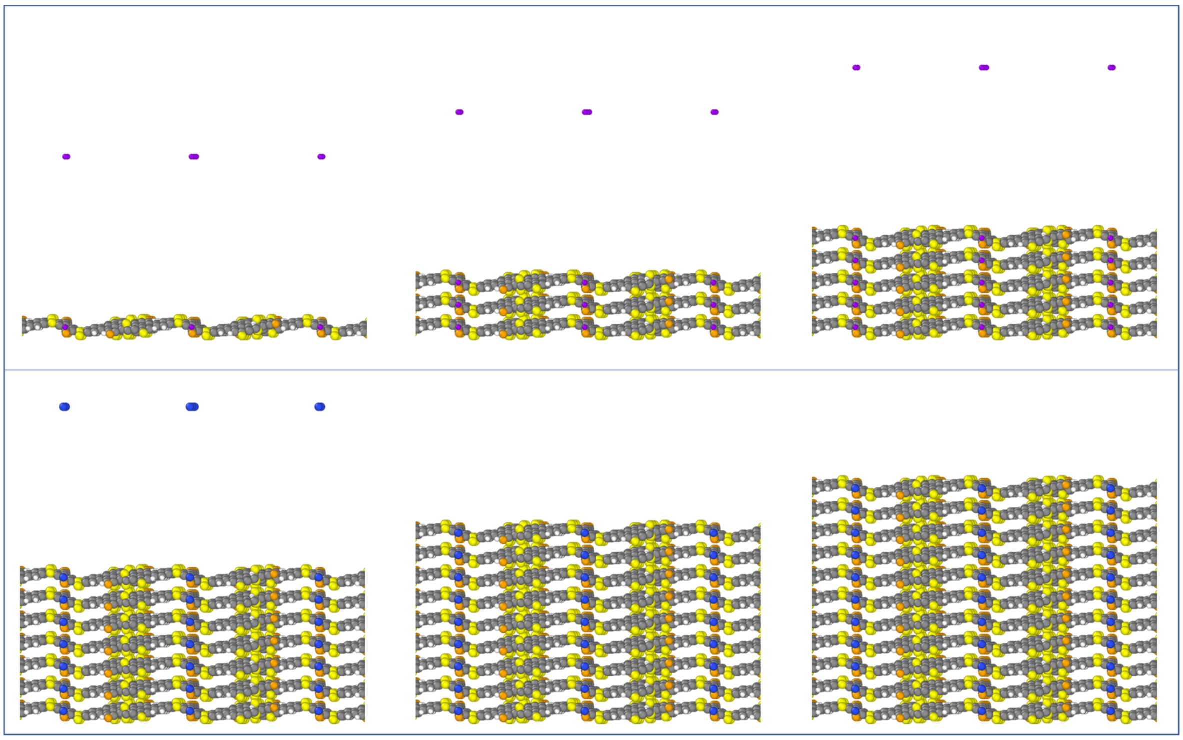

*Figure S2: Side views of the COF stacks of varying thickness (from 1 to 11 layers); the structures are periodically repeated in horizontal direction; the atoms 30 Å above the top COF layers (not visible for the 9- and 11-layer systems) are the Ne atoms used to probe the vacuum energy in the calculations of the finite-thickness stacks. The vacuum gap between periodic replicas of the stacks in vertical direction had a thickness of 70 Å. (color code - dark grey:C in the COF, light grey: C in C60, yellow: S, orange: N, white: H; blue/purple: Ne)*

# S2. Additional methodological details

## S2.1 Computational approach

For the presented studies the FHI-aims code [2–5] was used employing periodic boundary conditions and the Perdew-Burke-Ernzerhof (PBE) functional [6,7], which was coupled to a revised variant of the many-body dispersion correction.[8] For the basis-set the FHI-aims default "tight" settings were employed, which are discussed in more detail below. A well converged 2×2×12 k-points grid was used for the bulk calculations. Convergence criteria for the self-consistency cycle were set to the default values for changes in the charge density ($10^{-5}$), the total energy ($10^{-6}$ eV), the forces ($10^{-4}$ eV·Å$^{-1}$). To determine the occupation of the Kohn-

Sham eigenstates a Gaussian broadening function with a width of $\sigma$ = 0.01 eV was used. The atomic ZORA approximation was used [9] to account for relativistic effects.

Local geometry optimizations were performed using the version of the Broyden-Fletcher-Shanno-Goldfarb optimization algorithm enhanced by the trust radius method [2], with a tolerance threshold of $10^{-2}$ eV·Å$^{-1}$. For the geometry optimizations of the bulk structures, we started from the structure described in the supporting information of [1]. For obtaining an eclipsed arrangement of the COF planes and for simplifying the pore structure, the angles between unit cell vectors $\mathbf{a_3}$ and $\mathbf{a_1}$ and $\mathbf{a_3}$ and $\mathbf{a_2}$ were then set to 90° and between $\mathbf{a_1}$ and $\mathbf{a_2}$ to 120°. These angles were kept fixed for most calculations, while the lengths of the unit cell vectors were optimized together with the atomic positions. For the sake of comparison, also fully optimized unit cells with a slightly serrated arrangement of the COF planes were studied. They were realized by starting from a slightly distorted geometry (not to get stuck in a saddle point geometry) by somewhat tilting the $\mathbf{a_3}$ vector and then fully optimizing all geometrical parameters including the angles between the unit cell vectors. As shown in section S4, this had essentially no impact on the effects in the focus of the current study. Therefore, all data presented in the main manuscript were obtained for eclipsed structures.

A peculiarity of the -$NH_2$ substituents is that due to its non-planar structure it features a non-vanishing component of the dipole moment parallel to the pore axis. Thus, to avoid the buildup of a significant potential gradient within the pore, neighboring -$NH_2$ groups were rotated such that their dipole moments parallel to the pore compensated each other.

When increasing the distance between COF layers (c.f., for example Figure S1) the length of the $\mathbf{a_3}$ vector was increased by the specified value without further geometry optimizations and the k-grid was scaled accordingly. For building COF stacks, first a supercell containing the given number of layers was constructed. Subsequently, the length of the $\mathbf{a_3}$ vector of the supercell was increased by 70Å (unless specified differently) in order to decouple periodic replicas of the stacks and to be able to probe the vacuum level at a sufficiently large distance from the stack (cf. section S6). To decouple the stacks electrostatically, a self-consistently determined dipole layer was included in this vacuum gap.[10,11] Consequently, in the direction perpendicular to the COF layers, only the $\Gamma$-point was considered for calculating the electronic structure. Also for the stacks no further geometry optimizations were performed. Details on how the structures containing C60 molecules were generated are provided in section S8. In short, the unit cells of optimized eclipsed COF structures were tripled in $\mathbf{a_3}$ direction, $C_{60}$ molecules were independently optimized in the resulting unit cells (without the COF being present) and were then placed in the centers of the pores without any further geometry optimizations.

Plots of the MOF geometries and isovalue plots of the electrostatic energy were produced using OVITO [12]; the electrostatic energy in the cross-sections in Figure 3 were plotted using VESTA.[13] Marvinsketch was used for drawing, chemical structures, ChemAxon (https://www.chemaxon.com)

### S2.2 Determining the electrostatic energy

The electrostatic energy either in the pores or in the vacuum region plays a crucial role in the present study. Two strategies were used for its determination (which yielded consistent results): For all isovalue plots and for the cross-sections in Figure 3, the Hartree potentials was simply written out on a grid (in a so-called cube-file). For determining the electrostatic energy at specific locations, it was exploited that core-level energies serve as a direct probe for the local electrostatic potential with electrostatic core-level shifts superimposed on possible chemical shifts.[14] To eliminate chemical shifts, a Ne atom was used as inert probe particle, which was placed inside the channels (far away from the pore walls) or in vacuum 30 Å above the topmost layer finite-thickness COFs (for distance convergence see section S4). In the present study only relative core-level shifts are relevant. These are found to be fully consistent with the calculated differences in Hartree energies.

As illustrated, e.g., in Figure S5, this probe particle had no relevant impact on the electrostatic energy, but comparing the core-level shifts of the Ne-1s states of atoms in different positions provided a straightforward tool for determining variations in the electrostatic energies. This strategy hints towards a possible direct experimental strategy for determining the electrostatic energy shifts discussed in the present paper via x-ray photoelectron spectroscopy experiments, although in experimental practice Ne atoms might not be the ideal probe particles for that purpose and should be replaced by conventional guest molecules. As only core-level shifts are relevant in the present context, the $Ne_{1s}$ core-level energies were determined using the initial-state approach.[15–18] In fact, considering differences screening effects via final-state approaches would have even obscured the information on changes in the electrostatic energy.

### S2.3 Details on the employed basis set(s)

In spherical coordinates (r, $\Theta$, $\Phi$) relative to a given atomic center The basis functions employed in the FHI-aims simulations have the format[*]

[*] As described in the FHI-aims manual, version January 23, 2017.

$$\Phi(r) = \frac{u(r)}{r} * Y_{lm}(\Theta, \Phi)$$

with u(r) being a predefined radial function and the $Y_{lm}$ being spherical harmonics. The details of the functions (including information on, e.g., integration grids, etc.) are contained in so-called *species_defaults* file provided by FHI-aims. Typically, for all calculations the predefined "tight" basis sets were employed (without any further adjustments). "Intermediate" basis sets were employed only for certain convergence tests described in sections S3 and S5.

*Table S2. Basis functions that have been used for all calculations performed with FHI-aims. The abbreviations read as follows: X(nl, z), where X describes the type of basis function where H stands for hydrogen-like functions and ionic for a free-ion like radial function. The parameter n stands for the main/radial quantum number, l denotes the angular momentum quantum number (s, p, d, f, …), and z denotes an effective nuclear charge, which scales the radial function in the defining Coulomb potential for the hydrogen-like function. In the case of free-ion like radial functions, z specifies the onset radius of the confining potential. If auto is specified instead of a numerical value, the default onset is used.*

| | H | C | N | S | F |
|---|---|---|---|---|---|
| Minimal | valence (1s, 1.0) | valence (2s, 2.0) | valence (2s, 2.0) | valence (3s, 2.0) | valence (2s, 2.0) |
| | ion_occ (1s, 0.5) | valence (2p, 2.0) | valence (2p, 3.0) | valence (3p, 4.0) | valence (2p, 5.0) |
| | | ion_occ (2s, 2.0) | ion_occ (2s, 1.0) | ion_occ (3s, 1.0) | ion_occ (2s, 1.0) |
| | | ion_occ (2p, 2.0) | ion_occ (2p, 2.0) | ion_occ (3p, 3.0) | ion_occ (2p, 4.0) |
| First tier | H(2s, 2.1) | H(2p, 1.7) | H(2p, 1.8) | Ionic (3d, auto) | H(2p, 1.7) |
| | H(2p, 3.5) | H(3d, 6) | H(3d, 6.8) | H(2p, 1.8) | H(3d, 4.7) |
| | | H(2s, 4.9) | H(3s, 5.8) | H(4f, 7) | H(3s, 6.8) |
| | | | | Ionic (3s, auto) | |
| Second tier | H(1s, 0.85) | H(4f, 9.8) | H(4f, 10.8) | H(4d, 6.2) | H(4f, 11.2) |
| | H(2p, 3.7) | H(3p, 5.2) | H(3p, 5.8) | H(5g, 10.8) | Ionic (2p,auto) |
| | H(2s, 1.2) | H(3s, 4.3) | H (1s, 0.8) | | H (1s, 0.75) |
| | H(3d, 7.0) | H(5g, 14.4) | H(5g, 16) | | H (4d, 8..8) |
| | | H(3d, 6.2) | H(3d, 4.9) | | H (5g, 16.8) |

## S2.4. Impact of basis-set and k-point sampling

To test the impact of the basis set, we calculated the properties of pCOF-CN stacks of varying thickness employing the default "tight" settings of FHIAims[2–5] using the basis set described in the previous section and employing a well converged 2×2×12 k-point grid for describing the electron density and compared the results to calculations employing "intermediate" settings/basis sets in combination with a 1×1×6 k-point grid. The obtained results for the differences in electrostatic energies between pore centers and the vacuum above the stacks, $E^{*elstat}_{pore-vac}$, as a function of the number of COF layers are compared in Figure S3. There is a rigid shift between the "intermediate" and the "tight" values (with the latter being smaller by ca. 0.1 eV). The obtained trends are, however, the same. We attribute this deviation to the notoriously slow convergence of dipole moments with basis-set size. Therefore, all calculations contained in this paper have been performed using "tight" settings/basis sets despite the fact that the largest considered systems (11 layer stacks of pCOF-CN and pCOG-$NH_2$) contain more than 1000 atoms in the unit cells. This, however, rendered further increasing the basis set size not advisable; considering the similar trend already obtained with the "intermediate" settings also suggests that this is not necessary. Thus, for all data contained in this publication safe the ones in Figure S3 and the tests in section S5, the default "tight" settings/basis sets in combination with the 2×2×12 k-point grid were used.

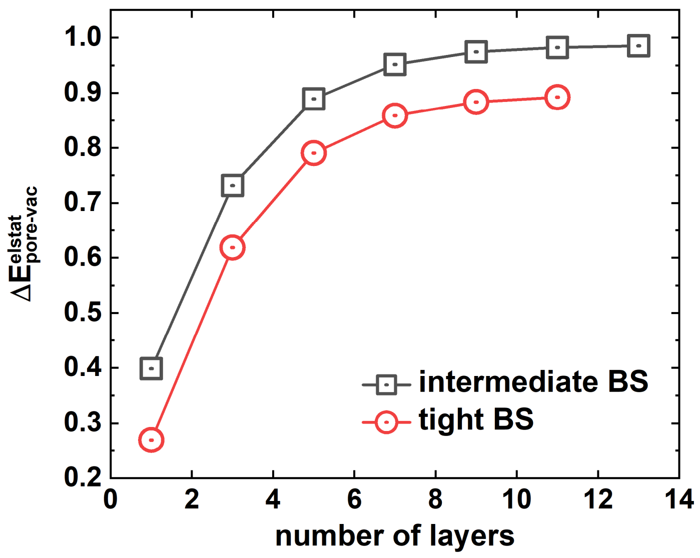


*Figure S3: Differences in electrostatic energies between pore centers and the vacuum above the stacks, $E^{*elstat}_{pore-vac}$, as a function of the number of COF layers for two different settings/basis sets ("intermediate" basis set and "tight" basis set†).*

† As described in the FHI-aims manual, version January 23, 2017.

## S3. Eclipsed vs. serrated structure

To assess the impact of the alignment of consecutive COF layers (eclipsed vs. staggered), the differences in electrostatic energies between pore centers and the vacuum above the stacks, $E_{pore-vac}^{*elstat}$, as a function of the number of COF layers was calculated for both structures (for details see section S1 and Table S1). Figure S4 shows that trends for $E_{pore-vac}^{*elstat}$ are essentially identical for both structures.

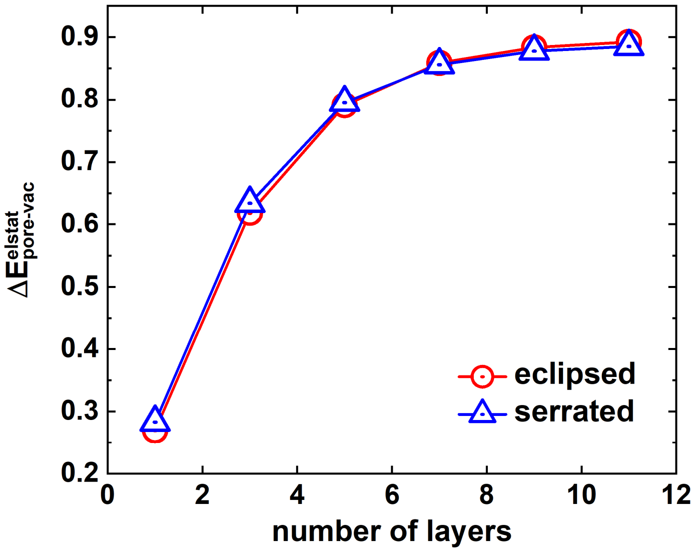


*Figure S3: Differences in electrostatic energies between pore centers and the vacuum above the stacks, $E_{pore-vac}^{*elstat}$, as a function of the number of COF layers for an eclipsed and a serrated arrangement of consecutive layers (for details on the structures see Table S1).*

## S4. Additional details on the determination of the vacuum level

To determine the electrostatic energy in vaccum above the COF stack in a way consistent with the determination of the electrostatic energy inside the pores, the 1s core-level energies of Ne atoms placed in the vacuum gap were used (see Section S1). This raises the question, how far the probing atoms need to be displaced from the topmost COF layer such that the energy of the vacuum level is no longer impacted by the potential variations caused by the COF stack. To assess that, we calculated the splitting between the Ne1s core-level and the valence band maximum of the COF as a function of the distance of the Ne probe from the center of the topmost COF layer. The result is shown in Figure S4 with essentially identical

trends obtained when plotting the $Ne_{1s}$ energy relative to the vacuum level above the slab or relative to the Fermi level. This plot reveals that the energy is very well converged for a distance of 30Å (in combination with a total width of the vacuum gap of 70 Å).

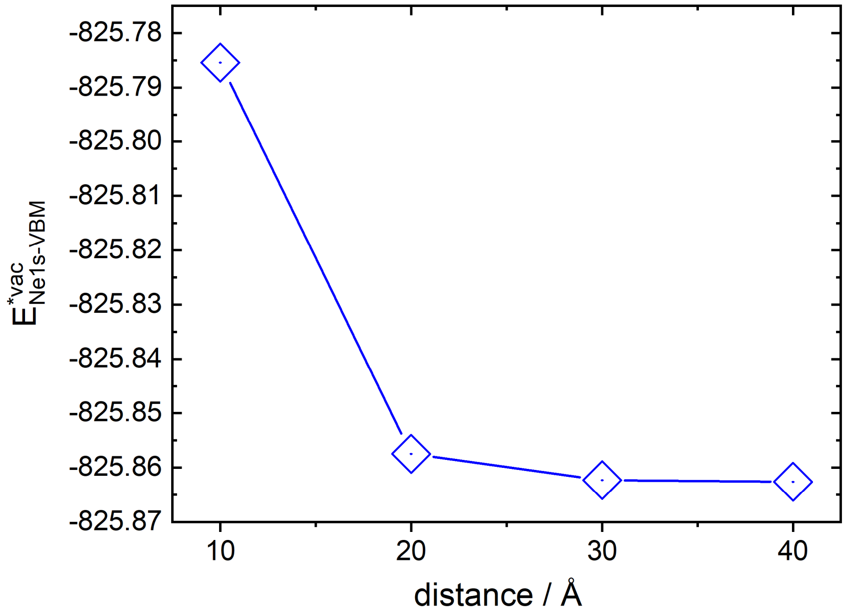


*Figure S4: Energy difference between the Ne1s core level of a neon atom in the vacuum region and the valence band edge of a 5-layer COF stack as a function of the distance of the Ne atom from the topmost COF layer. The width of the vacuum gap in these calculations was set to twice the distance of the Ne atom plus 10 Å. Apart from one of the datasets in Figure S3, these are the only data obtained with "intermediate" numerical settings/basis set and a 1×1×6 k-point grid.*

## S5. Core level binding energies and their shifts for the Ne atoms used as probes for the electrostatic potential

The data forming the basis for Figures 2(b) and (c) in the main manuscript are summarized in the following table.

*Table S3: Core-level energies of the 1s state of the Ne atoms used as probes for the electrostatic energy for the studied COFs as a function of the number of layers in the COF stack relative to the vacuum energy above the slab, $E_{Ne1s}$ (white and grey background). The numbers plotted with grey background refer to the Ne atoms placed as probes in vacuum 30 Å above the topmost COF layer. The core-level energies of the Ne atoms placed at the centers of the COF layers in the middle of the pores (white background), are also given relative to the energies for the atoms in vacuum, $\Delta E_{Ne1s}$ (green backgrounds). For the 11-layer stacks, these numbers are plotted in Figure 2(c). The numbers plotted in bold refer to the shift in core-level energy for the Ne atoms in the middle of the stacks. They are plotted in Figure 2(b) of the main manuscript.*

| **pCOF-CN** (eclipsed configuration) | | | | | | | | | | | |
|---|---|---|---|---|---|---|---|---|---|---|---|
| 1 layer | | 3 layers | | 5 layers | | 7 layers | | 9 layers | | 11 layers | |
| $E_{Ne1s}$ / eV | $\Delta E_{Ne1s}$ / eV | $E_{Ne1s}$ / eV | $\Delta E_{Ne1s}$ / eV | $E_{Ne1s}$ / eV | $\Delta E_{Ne1s}$ / eV | $E_{Ne1s}$ / eV | $\Delta E_{Ne1s}$ / eV | $E_{Ne1s}$ / eV | $\Delta E_{Ne1s}$ / eV | $E_{Ne1s}$ / eV | $\Delta E_{Ne1s}$ / eV |
| -831.72 | | -831.72 | | -831.72 | | -831.72 | | -831.72 | | -831.72 | |
| -831.45 | **0.269** | -831.20 | 0.521 | -831.15 | 0.565 | -831.15 | 0.571 | -831.15 | 0.572 | -831.15 | 0.572 |
| | | -831.19 | 0.532 | -831.14 | 0.577 | -831.13 | 0.583 | -831.13 | 0.583 | -831.13 | 0.583 |
| | | -831.10 | **0.619** | -830.98 | 0.735 | -830.97 | 0.752 | -830.96 | 0.754 | -830.96 | 0.754 |
| | | | | -830.98 | 0.740 | -830.96 | 0.757 | -830.96 | 0.759 | -830.96 | 0.759 |
| | | | | -830.93 | **0.791** | -830.88 | 0.836 | -830.87 | 0.842 | -830.87 | 0.843 |
| | | | | | | -830.88 | 0.838 | -830.87 | 0.844 | -830.87 | 0.844 |
| | | | | | | -830.86 | **0.859** | -830.84 | 0.875 | -830.84 | 0.877 |
| | | | | | | | | -830.84 | 0.876 | -830.84 | 0.878 |
| | | | | | | | | -830.83 | **0.884** | -830.83 | 0.889 |
| | | | | | | | | | | -830.83 | 0.890 |
| | | | | | | | | | | -830.82 | **0.892** |

| **pCOF-F** | | | | | |
|---|---|---|---|---|---|
| 1 layer | 3 layers | 5 layers | 7 layers | 9 layers | 11 layers |

| $E_{Ne1s}$ / eV | $\Delta E_{Ne1s}$ / eV | $E_{Ne1s}$ / eV | $\Delta E_{Ne1s}$ / eV | $E_{Ne1s}$ / eV | $\Delta E_{Ne1s}$ / eV | $E_{Ne1s}$ / eV | $\Delta E_{Ne1s}$ / eV | $E_{Ne1s}$ / eV | $\Delta E_{Ne1s}$ / eV | $E_{Ne1s}$ / eV | $\Delta E_{Ne1s}$ / eV |
|---|---|---|---|---|---|---|---|---|---|---|---|
| -831.72 | | -831.72 | | -831.72 | | -831.72 | | -831.72 | | -831.72 | |
| -831.69 | **0.024** | -831.68 | 0.039 | -831.68 | 0.042 | -831.68 | 0.042 | -831.67 | 0.042 | -831.67 | 0.042 |
| | | -831.68 | 0.040 | -831.67 | 0.042 | -831.67 | 0.043 | -831.67 | 0.043 | -831.67 | 0.043 |
| | | -831.67 | **0.045** | -831.66 | 0.052 | -831.66 | 0.053 | -831.66 | 0.053 | -831.66 | 0.053 |
| | | | | -831.66 | 0.052 | -831.66 | 0.053 | -831.66 | 0.053 | -831.66 | 0.053 |
| | | | | -831.66 | **0.056** | -831.66 | 0.058 | -831.66 | 0.058 | -831.66 | 0.058 |
| | | | | | | -831.66 | 0.058 | -831.66 | 0.059 | -831.66 | 0.059 |
| | | | | | | -831.66 | **0.059** | -831.66 | 0.060 | -831.66 | 0.060 |
| | | | | | | | | -831.66 | 0.060 | -831.66 | 0.060 |
| | | | | | | | | -831.66 | **0.061** | -831.66 | 0.061 |
| | | | | | | | | | | -831.66 | 0.061 |
| | | | | | | | | | | -831.66 | **0.061** |

| **pCOF-H** | | | | | | | | | | | |
|---|---|---|---|---|---|---|---|---|---|---|---|
| 1 layer | | 3 layers | | 5 layers | | 7 layers | | 9 layers | | 11 layers | |
| $E_{Ne1s}$ / eV | $\Delta E_{Ne1s}$ / eV | $E_{Ne1s}$ / eV | $\Delta E_{Ne1s}$ / eV | $E_{Ne1s}$ / eV | $\Delta E_{Ne1s}$ / eV | $E_{Ne1s}$ / eV | $\Delta E_{Ne1s}$ / eV | $E_{Ne1s}$ / eV | $\Delta E_{Ne1s}$ / eV | $E_{Ne1s}$ / eV | $\Delta E_{Ne1s}$ / eV |
| -831.79 | | -831.90 | | -831.96 | | -831.99 | | -832.00 | | -832.00 | |
| -831.72 | **-0.070** | -831.87 | **-0.185** | -831.95 | **-0.245** | -831.98 | **-0.271** | -832.00 | **-0.282** | -832.00 | **-0.285** |
| | | -831.87 | -0.157 | -831.94 | -0.228 | -831.98 | -0.264 | -832.00 | -0.282 | -832.00 | -0.284 |
| | | -831.72 | -0.155 | -831.89 | -0.227 | -831.95 | -0.263 | -831.98 | -0.266 | -832.00 | -0.284 |
| | | | | -831.89 | -0.175 | -831.95 | -0.235 | -831.98 | -0.266 | -832.00 | -0.279 |
| | | | | -831.72 | -0.172 | -831.89 | -0.234 | -831.95 | -0.266 | -831.98 | -0.279 |

| | | | | | | | | | | | |
|---|---|---|---|---|---|---|---|---|---|---|---|
| | | | | | | -831.89 | -0.177 | -831.95 | -0.236 | -831.98 | -0.267 |
| | | | | | | -831.72 | -0.174 | -831.90 | -0.235 | -831.95 | -0.266 |
| | | | | | | | | -831.89 | -0.178 | -831.95 | -0.236 |
| | | | | | | | | -831.72 | -0.174 | -831.90 | -0.235 |
| | | | | | | | | | | -831.89 | -0.178 |
| | | | | | | | | | | -831.72 | -0.175 |

| **pCOF-NH$_2$** | | | | | | | | | | | |
|---|---|---|---|---|---|---|---|---|---|---|---|
| 1 layer | | 3 layers | | 5 layers | | 7 layers | | 9 layers | | 11 layers | |
| $E_{Ne1s}$ / eV | $\Delta E_{Ne1s}$ / eV | $E_{Ne1s}$ / eV | $\Delta E_{Ne1s}$ / eV | $E_{Ne1s}$ / eV | $\Delta E_{Ne1s}$ / eV | $E_{Ne1s}$ / eV | $\Delta E_{Ne1s}$ / eV | $E_{Ne1s}$ / eV | $\Delta E_{Ne1s}$ / eV | $E_{Ne1s}$ / eV | $\Delta E_{Ne1s}$ / eV |
| -831.85 | | -832.05 | | -832.15 | | -832.20 | | -832.21 | | -832.22 | |
| -831.72 | **-0.131** | -832.00 | **-0.332** | -832.12 | **-0.436** | -832.18 | **-0.480** | -832.21 | **-0.497** | -832.22 | **-0.503** |
| | | -832.00 | -0.283 | -832.12 | -0.406 | -832.18 | -0.467 | -832.21 | -0.497 | -832.22 | -0.501 |
| | | -831.72 | -0.279 | -832.03 | -0.405 | -832.13 | -0.466 | -832.19 | -0.471 | -832.21 | -0.501 |
| | | | | -832.03 | -0.312 | -832.13 | -0.418 | -832.19 | -0.471 | -832.21 | -0.493 |
| | | | | -831.72 | -0.308 | -832.03 | -0.416 | -832.14 | -0.471 | -832.19 | -0.493 |
| | | | | | | -832.03 | -0.316 | -832.13 | -0.419 | -832.19 | -0.471 |
| | | | | | | -831.72 | -0.312 | -832.03 | -0.418 | -832.14 | -0.471 |
| | | | | | | | | -832.03 | -0.317 | -832.14 | -0.419 |
| | | | | | | | | -831.72 | -0.313 | -832.03 | -0.418 |
| | | | | | | | | | | -832.03 | -0.317 |
| | | | | | | | | | | -831.72 | -0.313 |

## S6. Isovalue plots of the electrostatic energies for selected periodic COFs and COF stacks

Figure 2(d) in the main manuscript shows an isovalue plot of the electrostatic energy for a pCOF-CN 7-layer stack for an isovalue of +0.7 eV relative to the vacuum level. It clearly illustrates the potential "pocket" that is formed inside the pore of the polar COF. In Figure S5 analogous plots are shown for different isovalues (for calculations containing also the Ne atoms used as probes for determining the electrostatic energy). These data show the edge effect of collective electrostatic shifts discussed in the context of Figure 2(c) in the main manuscript. They also illustrate that at least in the center of the stacks also for comparably large isovalues the isosurface extends towards the pore wall. Finally, they show that presence of the Ne atoms has virtually no impact on the collective electrostatic effects.

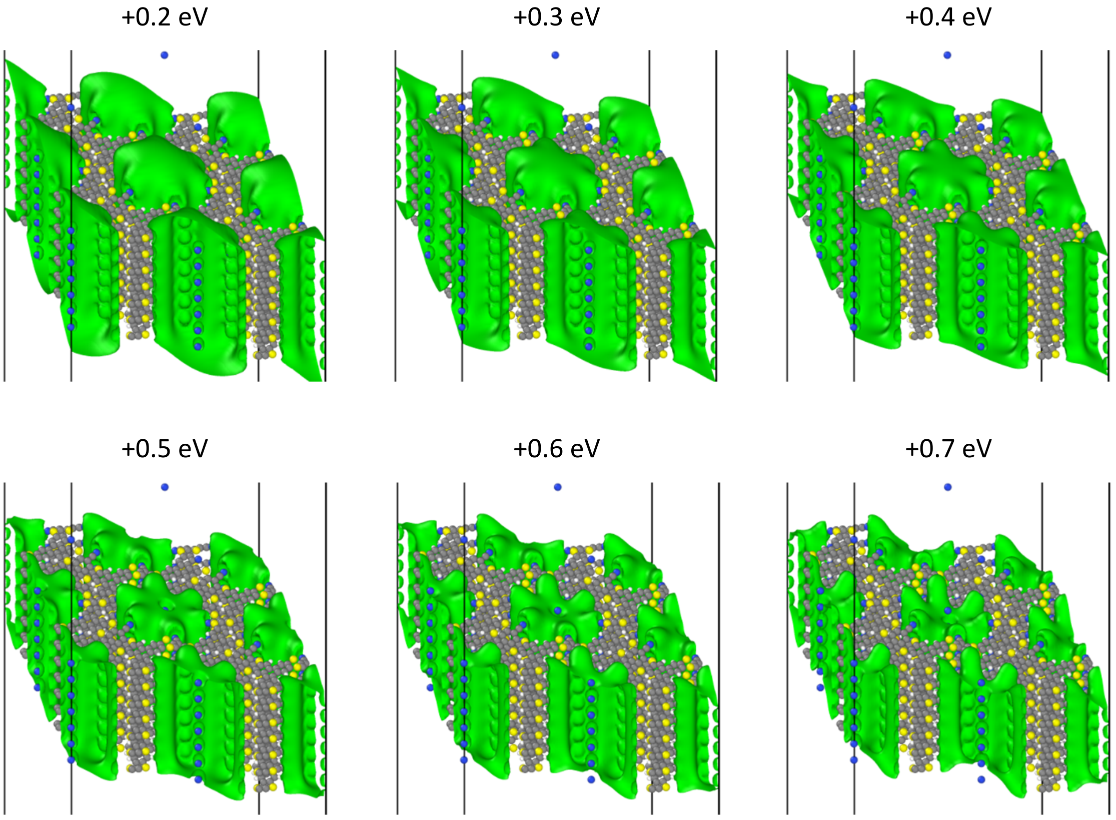


+0.8 eV +0.85 eV +0.9 eV

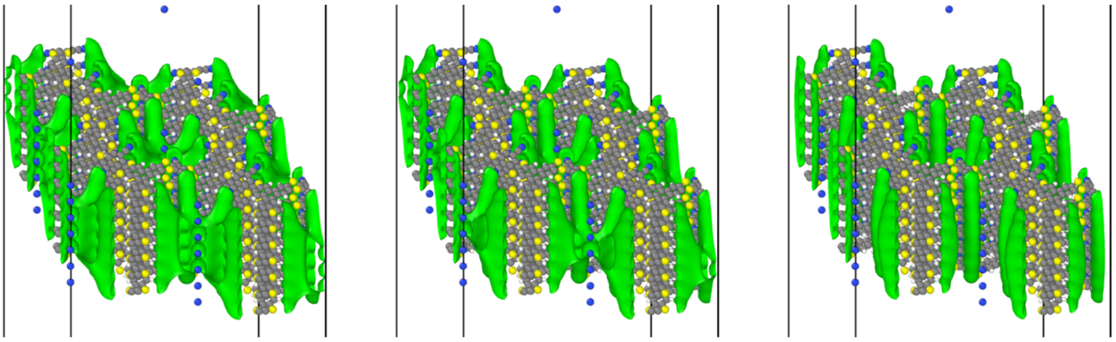

*Figure S5: Isovalue plots of the electrostatic (Hartree) energy of an electron for a 7-layer stack of pCOF-CN (eclipsed configuration) including the Ne atoms used to "measure" the potential distribution. The plotting range comprises a 2×2×1 supercell centered around a pore. The isovalues for the electrostatic energy relative to the vacuum level are specified above each plot.*

Plots similar to the above-discussed ones for the bulk COFs with artificially increased inter-layer distance are shown in Figure S6. The complication here is that (in contrast to the calculations on the stacks) the choice of a suitable energy reference relative to which the electrostatic energy is specified is less straightforward for bulk calculations (see also discussion in the main manuscript). Therefore, in Figure S6 the isovalue is chosen to be 5.95 eV above the position of the valence-band maximum of the COF. The value of 5.95 eV is chosen arbitrarily, but it allows illustrating the energy "pockets" formed in the pores in the region of the layers due to the combined fields caused by the polar substituents.

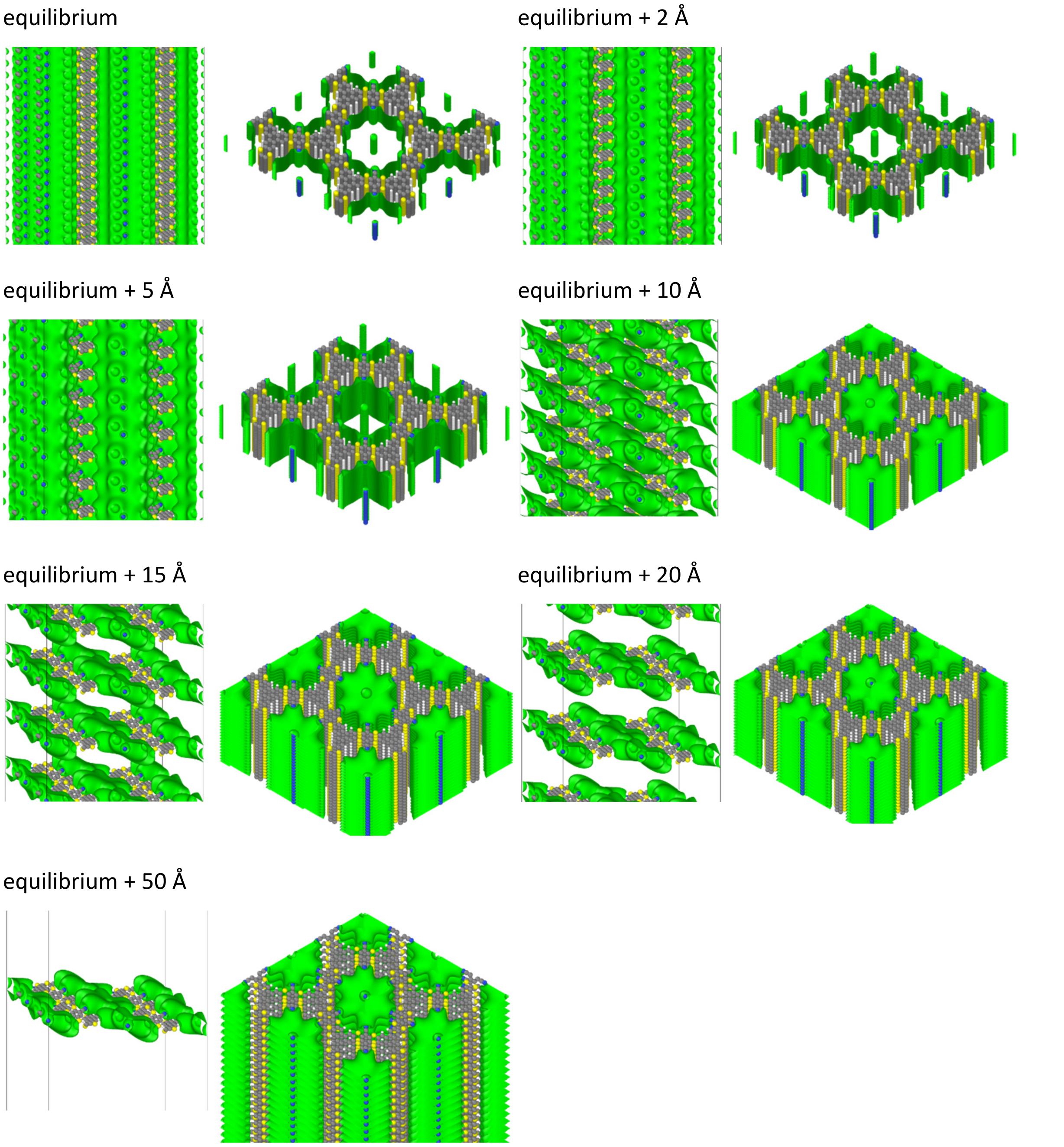


*Figure S6: Isovalue plots of the electrostatic (Hartree) energy of an electron for a DUT-177 COFs (eclipsed configuration) with an artificially increased inter-layer distance. The Ne atoms used to "measure" the potential distribution are included in the simulations and the plotting range comprises a 2×2×x supercells centered around a pore. The isovalue for the Hartree energy is set to the energy of the valence band maximum + 5.95 eV. Here one again encounters the problem of the ill-defined energy reference in bulk systems discussed in the main manuscript.*

Finally, Figure S7 contains top-views of isovalue plots of the electrostatic (Hartree) energy of pCOF-CN, and pCOF-$NH_2$ (this time not only for negative, but also for positive isovalues). Here one can see that in pCOF-CN the pockets of negative electrostatic energy extend towards the -CN substituents, while they appear to be "repelled" by the -CH groups. For pCOF-CN, negative electrostatic energies are exclusively found in the immediate vicinity of the atoms due to the fields generated by the nuclei. Conversely, for pCOF-$NH_2$ regions of negative electrostatic energy cover also the pores, while here positive values are found only in the immediate vicinity of the nitrogen atoms of the -NH2 groups.

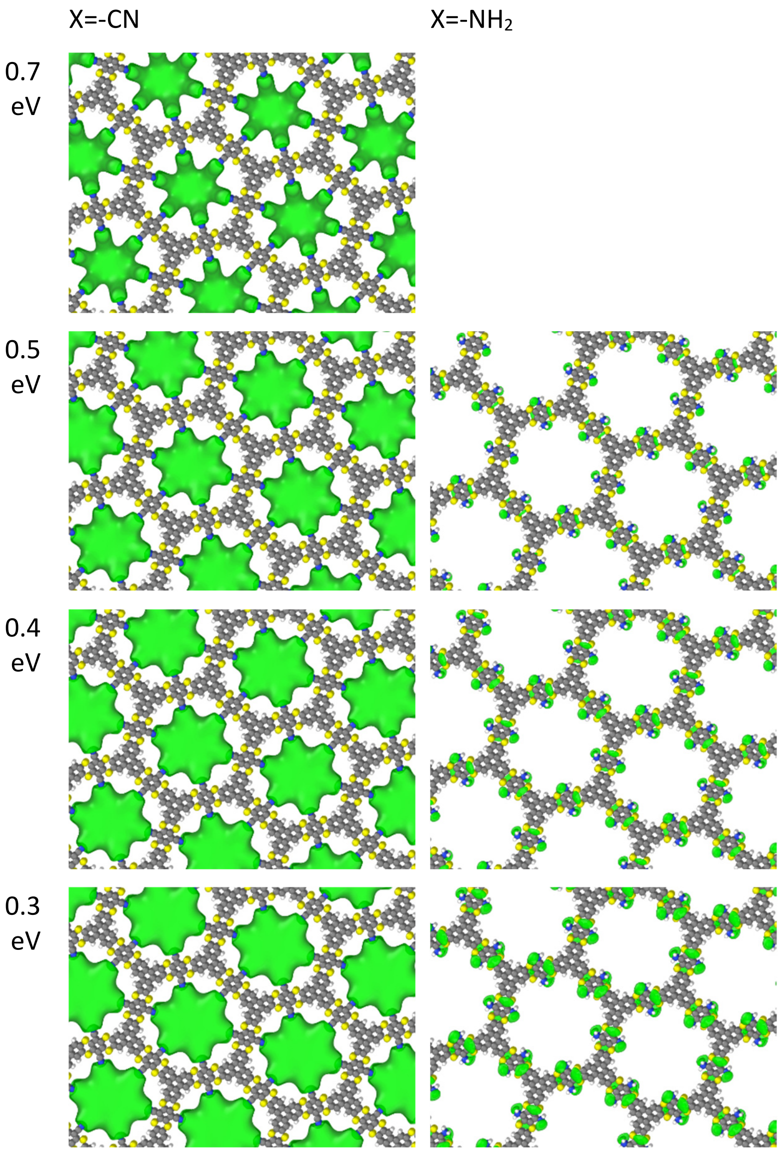

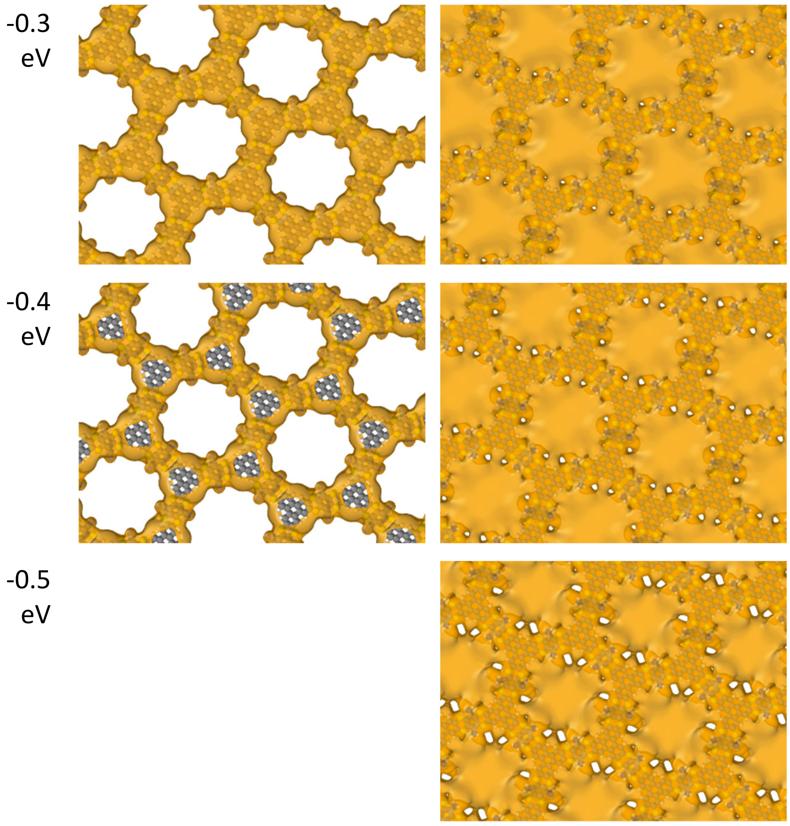


*Figure S7: Top view of the isovalue plots of the electrostatic (Hartree) energy of an electron for 7-layer stacks of pCOF-CN (eclipsed configuration) and pCOF-$NH_2$. The isovalues for the electrostatic energy relative to the vacuum level are specified in the left column.*

# S7. Additional information on the simulation of COFs containing $C_{60}$ molecules in the pores

A top-view of the structure of COFs containing C60 molecules in the pores is already contained in Figure 1 in the main manuscript. Figure S8a shows the unit-cell used in the simulations and Figure S8b contains also a side view of the system, which illustrates that there is one $C_{60}$ molecule per three layers of COF. This results in a distance of neighboring $C_{60}$ molecules of 4.6 Å, which is well beyond their van der Waals distance, but one $C_{60}$ molecule per two COF layers would result in a too tight packing. The geometry of the $C_{60}$ molecule was optimized in the resulting unit cell but without the COF being present (i.e., the $C_{60}$ geometry essentially corresponds to that of the isolated system. Moreover, the C60 molecule was placed

in the center of the pore. Thus, overall, $C_{60}$ molecules in the pores of the studied COFs might well adopt structures different from the studied one, which, very likely, will be incommensurate with the COF lattice. This deviation is, however, not a complication here as the simulations with C60 in the COF pores primarily serve the purpose of illustrating, how, in general, the collective electrostatic effects caused by the dipoles on the pore walls impact the level alignment between states in the COF and guest molecules. Considering that laterally the potential in the pores does not vary very much unless one gets very close to the pore walls, one would, in fact, also not expect significant changes in the level alignment also for a fully optimized $C_{60}$ geometry.

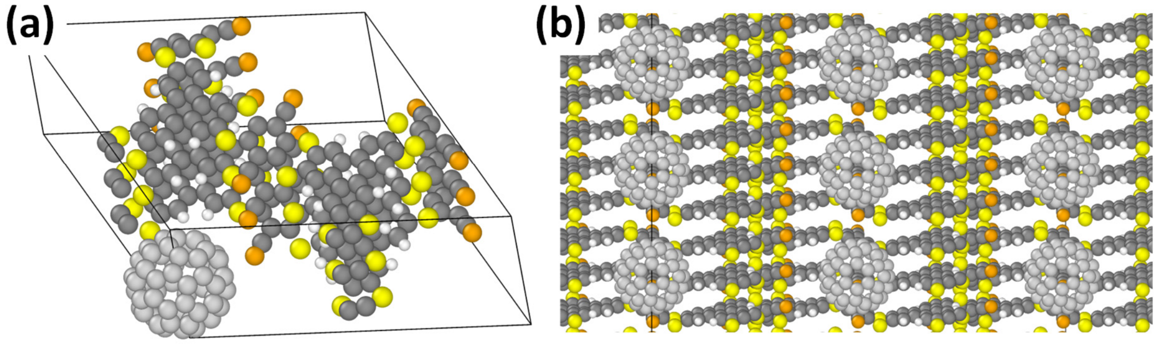


*Figure S8. (a) unit-cell for calculating periodic pCOF-CN in which the pore is filled by one C60 per three COF layers; (b) side view of extended structure. For top view see Figure 1 in the main manuscript.*

In the main manuscript (Figure 4(a) the substitution dependence of the energy of the valence-band maximum of the COF, $\mathrm{E}_{\mathrm{VBM}}^{*\mathrm{COF}}$,relative to the electrostatic energy in vacuum and in the pore is shown. Figure S9 contains an equivalent plot, but now for the conduction-band minimum, $\mathrm{E}_{\mathrm{CBM}}^{*\mathrm{COF}}$. While the absolute numbers are different, the trends for the two quantities are essentially the same.

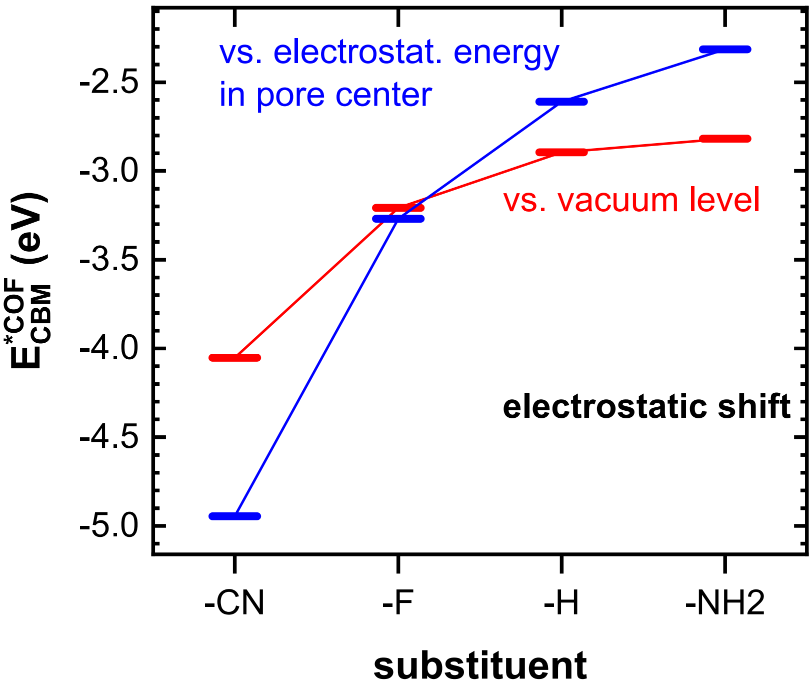


Figure S9. *Red bars: substitution dependence of the energy of the conduction-band minimum of the COF,* $E^{*COF}_{CBM}$*,relative to the electrostatic energy in vacuum, which is determined here 30 Å above the stack. This energy correlates the ionization process denoted by the red arrow in Figure 3(a). Within the single-electron picture it corresponds to the electron affinity of the COF. blue bars: substitution dependence of the energy of the valence-band maximum relative to the electrostatic energy in the center of the pore of the middle COF layer of a COF stack (process represented by the blue arrow in Figure 3(a)). The data here have been obtained for the 11-layer stack, for which the electrostatic energy in the central COF layer has essentially converged to the bulk value (see Figure 2b, main manuscript). The area shaded in green corresponds to the shift due to the collective electrostatic modification of the potential inside the pore.*

## References


(1) Haldar, S.; Wang, M.; Bhauriyal, P.; Hazra, A.; Khan, A. H.; Bon, V.; Isaacs, M. A.; De, A.; Shupletsov, L.; Boenke, T.; Grothe, J.; Heine, T.; Brunner, E.; Feng, X.; Dong, R.; Schneemann, A.; Kaskel, S. Porous Dithiine-Linked Covalent Organic Framework as a Dynamic Platform for Covalent Polysulfide Anchoring in Lithium–Sulfur Battery Cathodes. *J. Am. Chem. Soc.* **2022**, *144* (20), 9101–9112. https://doi.org/10.1021/jacs.2c02346.

(2) Blum, V.; Gehrke, R.; Hanke, F.; Havu, P.; Havu, V.; Ren, X.; Reuter, K.; Scheffler, M. Ab Initio Molecular Simulations with Numeric Atom-Centered Orbitals. *Computer Physics Communications* **2009**, *180* (11), 2175–2196. https://doi.org/10.1016/j.cpc.2009.06.022.

(3) Havu, V.; Blum, V.; Havu, P.; Scheffler, M. Efficient O(N) Integration for All-Electron Electronic Structure Calculation Using Numeric Basis Functions. *Journal of Computational Physics* **2009**, *228* (22), 8367–8379. https://doi.org/10.1016/j.jcp.2009.08.008.

(4) Marek, A.; Blum, V.; Johanni, R.; Havu, V.; Lang, B.; Auckenthaler, T.; Heinecke, A.; Bungartz, H.-J.; Lederer, H. The ELPA Library: Scalable Parallel Eigenvalue Solutions for Electronic Structure Theory and Computational Science. *J. Phys.: Condens. Matter* **2014**, *26* (21), 213201. https://doi.org/10.1088/0953-8984/26/21/213201.

(5) Yu, V. W.; Corsetti, F.; García, A.; Huhn, W. P.; Jacquelin, M.; Jia, W.; Lange, B.; Lin, L.; Lu, J.; Mi, W.; Seifitokaldani, A.; Vázquez-Mayagoitia, Á.; Yang, C.; Yang, H.; Blum, V. ELSI: A Unified Software Interface for Kohn–Sham Electronic Structure Solvers. *Computer Physics Communications* **2018**, *222*, 267–285. https://doi.org/10.1016/j.cpc.2017.09.007.

(6) Perdew, J. P.; Burke, K.; Ernzerhof, M. Generalized Gradient Approximation Made Simple. *Phys. Rev. Lett.* **1996**, *77* (18), 3865–3868. https://doi.org/10.1103/PhysRevLett.77.3865.

(7) Perdew, J. P.; Burke, K.; Ernzerhof, M. Generalized Gradient Approximation Made Simple [Phys. Rev. Lett. 77, 3865 (1996)]. *Phys. Rev. Lett.* **1997**, *78* (7), 1396–1396. https://doi.org/10.1103/PhysRevLett.78.1396.

(8) Hermann, J.; Tkatchenko, A. Density Functional Model for van Der Waals Interactions: Unifying Many-Body Atomic Approaches with Nonlocal Functionals. *Phys. Rev. Lett.* **2020**, *124* (14), 146401. https://doi.org/10.1103/PhysRevLett.124.146401.

(9) Lenthe, E. van; Baerends, E. J.; Snijders, J. G. Relativistic Regular Two-component Hamiltonians. *J. Chem. Phys.* **1993**, *99* (6), 4597–4610. https://doi.org/10.1063/1.466059.

(10) Neugebauer, J.; Scheffler, M. Adsorbate-Substrate and Adsorbate-Adsorbate Interactions of Na and K Adlayers on Al(111). *Phys. Rev. B* **1992**, *46* (24), 16067–16080. https://doi.org/10.1103/PhysRevB.46.16067.

(11) Freysoldt, C.; Eggert, P.; Rinke, P.; Schindlmayr, A.; Scheffler, M. Screening in Two Dimensions: $GW$ Calculations for Surfaces and Thin Films Using the Repeated-Slab Approach. *Phys. Rev. B* **2008**, *77* (23), 235428. https://doi.org/10.1103/PhysRevB.77.235428.

(12) Stukowski, A. Visualization and Analysis of Atomistic Simulation Data with OVITO–the Open Visualization Tool. *Modelling and Simulation in Materials Science and Engineering* **2010**, *18* (1), 015012. https://doi.org/10.1088/0965-0393/18/1/015012.

(13) Momma, K.; Izumi, F. VESTA 3 for Three-Dimensional Visualization of Crystal, Volumetric and Morphology Data. *J Appl Cryst* **2011**, *44* (6), 1272–1276. https://doi.org/10.1107/S0021889811038970.

(14) Taucher, T. C.; Hehn, I.; Hofmann, O. T.; Zharnikov, M.; Zojer, E. Understanding Chemical versus Electrostatic Shifts in X-Ray Photoelectron Spectra of Organic Self-Assembled Monolayers. *J. Phys. Chem. C* **2016**, *120* (6), 3428–3437. https://doi.org/10.1021/acs.jpcc.5b12387.

(15) Bagus, P. S.; Ilton, E. S.; Nelin, C. J. The Interpretation of XPS Spectra: Insights into Materials Properties. *Surface Science Reports* **2013**, *68* (2), 273–304. https://doi.org/10.1016/j.surfrep.2013.03.001.
(16) Perdew, J. P.; Norman, M. R. Electron Removal Energies in Kohn-Sham Density-Functional Theory. *Phys. Rev. B* **1982**, *26* (10), 5445–5450. https://doi.org/10.1103/PhysRevB.26.5445.
(17) Stowasser, R.; Hoffmann, R. What Do the Kohn−Sham Orbitals and Eigenvalues Mean? *J. Am. Chem. Soc.* **1999**, *121* (14), 3414–3420. https://doi.org/10.1021/ja9826892.
(18) Chong, D. P.; Gritsenko, O. V.; Baerends, E. J. Interpretation of the Kohn–Sham Orbital Energies as Approximate Vertical Ionization Potentials. *J. Chem. Phys.* **2002**, *116* (5), 1760–1772. https://doi.org/10.1063/1.1430255.
